# Transcriptome Profiling of Tomato Fruit Development Reveals Transcription Factors Associated with Ascorbic Acid, Carotenoid and Flavonoid Biosynthesis

**DOI:** 10.1371/journal.pone.0130885

**Published:** 2015-07-02

**Authors:** Jie Ye, Tixu Hu, Congmei Yang, Hanxia Li, Mingze Yang, Raina Ijaz, Zhibiao Ye, Yuyang Zhang

**Affiliations:** Key Laboratory of Horticultural Plant Biology, Ministry of Education, Huazhong Agricultural University, Wuhan, China; University Paris South, FRANCE

## Abstract

Tomato (*Solanum lycopersicum*) serves as a research model for fruit development; however, while it is an important dietary source of antioxidant nutrients, the transcriptional regulation of genes that determine nutrient levels remains poorly understood. Here, the transcriptomes of fruit at seven developmental stages (7, 14, 21, 28, 35, 42 and 49 days after flowering) from two tomato cultivars (Ailsa Craig and HG6-61) were evaluated using the Illumina sequencing platform. A total of 26,397 genes, which were expressed in at least one developmental stage, were detected in the two cultivars, and the expression patterns of those genes could be divided into 20 groups using a K-mean cluster analysis. Gene Ontology term enrichment analysis indicated that genes involved in RNA regulation, secondary metabolism, hormone metabolism and cell wall metabolism were the most highly differentially expressed genes during fruit development and ripening. A co-expression analysis revealed several transcription factors whose expression patterns correlated with those of genes associated with ascorbic acid, carotenoid and flavonoid biosynthesis. This transcriptional correlation was confirmed by agroinfiltration mediated transient expression, which showed that most of the enzymatic genes in the ascorbic acid biosynthesis were regulated by the overexpression of each of the three transcription factors that were tested. The metabolic dynamics of ascorbic acid, carotenoid and flavonoid were investigated during fruit development and ripening, and some selected transcription factors showed transcriptional correlation with the accumulation of ascorbic acid, carotenoid and flavonoid. This transcriptome study provides insight into the regulatory mechanism of fruit development and presents candidate transcription factors involved in secondary metabolism.

## Introduction

Fleshy fruit development and ripening involve the coordinated regulation of numerous metabolic pathways that influence fruit nutrient levels and quality [1]. Cell division followed by cell expansion, resulting in the formation of large vacuolated cells, eventually leads to an increase in fruit weight and is accompanied by the accumulation of nutrients [2]. The subsequent increase in the accumulation of carotenoids, flavonoids, vitamins and other compounds that typically occurs during fruit ripening enhances both the sensory and nutritional quality of fruits; two parameters that are beneficial for human diet and that promote seed dispersal [3, 4].

The nutrient accumulation during fruit development and ripening is the final outcome of a complex set of integrated processes involving gene expression, protein translation and metabolic accumulation [5]. For most metabolites, such as carotenoids, flavonoids and ascorbic acid, the biosynthetic pathways have been well characterized in tomato; however, the underlying regulatory mechanisms remain largely unknown [6]. Transcriptome analysis has revealed fluctuations in the expression of genes involved in various metabolic pathways during fruit development and ripening together with diverse patterns of metabolite accumulation [7]. For example, the transcript abundance of genes encoding enzymes involved in ascorbic acid biosynthesis shows diurnal fluctuations that are influenced by light, indicating the existence of regulatory networks that are affected by external factors [8]. Indeed, these metabolites accumulate to varying degrees during fruit development and have been reported to be affected by environmental factors [9]. The fact that fruit nutrient status is subject to environmental regulation in addition to being developmentally regulated indicates that multiple transcription factors or regulators may act to control their biosynthesis [10].

Studies of transcription factors related to biosynthetic genes may help dissect the regulatory machinery that influences metabolite accumulation. Several such regulatory genes have been reported to be associated with carotenoid metabolism: for example, *RAP2*.*2* and *PIF1* have been shown to modulate carotenoid accumulation by transcriptionally regulating *PSY* in Arabidopsis [11, 12]. By comparative analysis with tomato genome, six of nineteen carotenoid-associated transcription factors were differentially expressed during fruit development and ripening in watermelon [13]. In tomato, several transcription factors, such as CNR, RIN, TAGL1 and SGR have been shown to be involved in regulating ripening and, thus, fruit carotenoid accumulation [14–17], while CSN5B, AtERF98, ABI4, and AMR1 are known to regulate ascorbic acid levels in the context of both stress responses and growth regulation [10, 18–20]. Ascorbic acid levels have also been shown to be modified via transcriptional regulation of the biosynthetic pathway in high-pigment tomato, a mutant in light signal transduction [21]. Moreover, it has been reported that the MYB transcription factor, *Sl*MYB12 modulates the expression of flavonoid biosynthetic genes in tomato fruits [22] and the flavonoid biosynthetic pathway is activated in tomato fruit by the transcription factors Del and Ros1 [23].

All these regulators or transcription factors have been characterized based on their respective mutants, but another strategy is to use transcriptome analysis to identify potential transcription factors associated with specific aspects of fruit development and biosynthetic pathways [24]. In one study, differential expression profiling identified 72 signal transduction or transcriptional factors genes that are potentially involved in tomato fruit development and ripening [25] and, in parallel, genes involved in anthocyanin biosynthesis have been found to be up-regulated during the fruit ripening processes, concurrent with color change and fruit development [26]. In this regard, transcript co-expression analysis is a potentially valuable strategy to link of transcription factors and structural genes. Such a relationship was reported for the flavonoid biosynthetic pathway, where gene expression studies combined with genetic mapping and segregation analysis suggested that *SlMYB12* is a likely candidate for the *y* locus, which is responsible for flavonoid accumulation [22].

In this current study, RNA-seq combined with metabolism analysis was used to investigate global dynamic changes in gene expression and metabolite accumulation during the development and ripening of fruit from two tomato cultivars, Ailsa Craig (AC) and HG6-61. The two cultivars originated from different area and showed different maturity progress, AC is an English variety with precocity, while HG6-61was an elite line with late-maturity from China. Co-expression analysis of transcription factors and structural genes involved in ascorbic acid, carotenoid and flavonoid biosynthesis was performed, in order to identify putative transcription factors that regulate these biosynthetic pathways. Finally, an agroinfiltration assay was also used to investigate the effect of the candidate transcription factors on these specific metabolic pathways.

## Materials and Methods

### Plant material and growth conditions

Tomato plants (*Solanum lycopersicum*; cultivars AC and HG6-61) were grown in the same greenhouse at the National Center for Vegetable Improvement (Central China) during the spring season. Plant growth and cultivation was carried out according to commercial practices with a day/night temperature of 28/20°C. Plants were pruned so that fruits were on one vine per plant. To collect fruit samples from various developmental stages, flowers were tagged when fully opened (anthesis) and fruits were harvested from 5 individual plants of each genotype at 7, 14, 21, 28, 35, 42 and 49 days after flowering (DAF). In order to ensure the uniformity of the fruit samples at each stage, harvested fruits were visually inspected externally and internally (e.g. size, shape, pigmentation, seed development and locular jelly formation), and only fruits that were developmentally equivalent were used in the subsequent analysis [5]. The fruit pericarp at each stage were mixed, snap-frozen in liquid nitrogen and kept at -80°C until further analysis. For agroinfiltration, greenhouse grown AC plants were grown at 28°C/20°C (day/night) with a 16 h photoperiod in 10 cm (diameter) plastic pots and fruits left on the plant until the breaker stage were used for agroinfiltration [27].

### RNA extraction and RNA-seq

The AC and HG6-61 fruits harvested at 7, 14, 21, 28, 35, 42 and 49 DAF were frozen in liquid nitrogen and kept at -80°C until use. Total RNA was extracted using a ZP411-2 GREENspin RNA quick extraction kit (ZOMANBIO, Beijing), excluding polyphenols and polysaccharides from the sample according to the manufacturer’s instructions. Total RNA were then sent to ABlife Wuhan where the libraries were produced and sequenced using Illumina's Genome AnalyzerIIx. Fruit sampling and RNA-seq from two cultivars AC and HG6-61 were carried out in parallel as two biological replicates. The sequencing data can be accessed at the website: http://www.ncbi.nlm.nih.gov/geo/query/acc.cgi?acc=GSE64981. Raw sequences were filtered to remove the 3’ adaptor sequence, low-quality reads (reads containing sequencing Ns > 5) and short reads (<16 nt) and the resulting sets of clean reads were used for the following analysis, as described previously [28]. All cleaned reads were mapped to contig assemblies using the Tophat mapping algorithm with the version 2.0.4 (http://tophat.cbcb.umd.edu/) allowing no more than 2-nucleotide mismatches. Clean reads that mapped to the genome sequences (SL2.40 version) of *Solanum lycopersicum* downloaded from SOL Genomics Network database (SGN, http://solgenomics.net/organism/Solanum_lycopersicum/genome). The multiple aligned reads were then filtered by tophat software and the remaining clean reads were designated as unambiguous clean reads. The number of unambiguous clean reads for each gene was calculated and then normalized to reads per kilobase of gene per million reads (RPKM), a standard unit to calculate UniGene expression [29]. The software edgeR was used to perform differential expression analysis [30]. edgeR can be used to analyze the difference in expression between two or more samples and indices of fold change (Log_2_ ratio) and p-value (false discovery rate) provide an indication of whether a gene is differentially expressed. Here, genes with a p-value < 0.01 and a Log_2_ ratio > 2.0 or < -2.0 were considered to be differentially expressed. The differentially expressed tomato genes extracted from ITAG2.4_proteins.fasta (ftp://ftp.solgenomics.net/genomes/Solanum_lycopersicum/annotation/ITAG2.4_release/) were used as query to identify *Arabidopsis thaliana* homologs (TAIR9 version) using an e-value of 1×e^-5^ by blastp (http://blast.ncbi.nlm.nih.gov/Blast.cgi). Finally, DAVID software was applied to perform a GO enrichment analysis of the annotated genes, as previously described [31].

### qRT-PCR

The expression pattern of selected differentially expressed genes identified in the RNA-seq analysis was validated by qRT-PCR. The expression abundance of biosynthetic genes following agroinfiltration of fruits (see below) was also investigated by qRT-PCR. The sequences of the primer pairs (designed using Primer Premier 3.0 [http://frodo.wi.mit.edu/primer3]) are listed in S1 Table. The cDNA synthesis and qRT-PCR steps were performed as previously described [32].

### Ascorbic acid, carotenoid and flavonoid extraction and HPLC analysis

Ascorbic acid extraction and HPLC analysis were carried out as described previously [33]. Briefly, samples were ground under liquid nitrogen and homogenised in 5 mL of cold 0.1% (w/v) metaphosphoric acid. The homogenate was then centrifuged at 12,000 g for 10 min at 4°C. The supernatant was filtered through a Millipore membrane (0.22 μm) to measure reduced ascorbate and an aliquot of 300 μL was incubated with 300 μL 50 mM dithiothreitol for 15 min at room temperature to measure total ascorbate. Then, the extracts were analyzed by HPLC using an SB-aq column (Agilent) eluted with acetate buffer (0.2 mol L^-1^ pH 4.5) at a flow rate of 1.0 mL min^-1^. Elutes were detected at 254 nm, and a standard curve from 2 to 40 μg mL^-1^ ascorbic acid was obtained.

Carotenoids were extracted and analyzed by HPLC as described previously [17]. Samples were ground into powder after freezing in liquid nitrogen. Carotenoids were eluted with methanol-methyl *tert*-butyl ether-H_2_O(81: 15: 4, v/v/v; eluent A) and methanol-methyl *tert*-butyl ether-H_2_O (10: 90: 4, v/v/v; eluent B) by a C_30_ carotenoid column (150x4.6 mm i.d., 3μm) from Waters. The linear gradient program was performed as follows: initial condition was 100% A to 100% B in 90 min, and back to the initial condition for re-equilibration. Analysis was conducted under subdued light to avoid carotenoid degradation during analysis. HPLC-grade β-carotene, lycopene, phytoene and lutein standards were obtained from Sigma (St Louis, MO, USA).

Flavonoids were extracted from 100 mg freeze-dried samples using 80% methanol which contains 0.1mg/L lidocaine as a quantification standard. The mixture was extracted for 12 h at 4°C. Flavonoids were analyzed and identified using a QToF 6520 mass spectrometer (Agilent Technologies, Palo Alto, CA, USA) coupled to a 1200 series Rapid Resolution HPLC system by a method modified from that described by [34]. 20μL of sample extract was loaded onto a Zorbax StableBond C18 1.8 μm, 2.1x100 mm reverse-phase analytical column (Agilent Technologies). Mobile phase A was 0.1% formic acid in water and mobile phase B was acetonitrile with 0.1% formic acid. The following gradient was used: 0 min-5% B; 20 min-95% B; 22 min-95%B; 22.1 min-10% B; 28 min-5% B. The flow rate was 0.3 mL min^-1^ and the column temperature was held at 35°C for the duration. The source conditions for electrospray ionization were as follows: gas temperature was 350°C with a drying gas flow rate of 10L min^-1^ and a nebulizer pressure of 55 psig. The capillary voltage was 3.5 kV in positive ion mode. The fragmentor voltage was 135V and skimmer 65V. Scanning was performed using the auto MS/MS function at 2 scans s^-1^ with a sloped collision energy of 3.5V/100 Da with an offset of 5V. Flavonoids were quantified by calculating the area of each individual peak and comparing this to internal standard.

### Correlation analysis of structural genes and transcription factors

A correlation analysis of structural genes and transcription factors was carried out to identify transcription factors that were co-expressed with the enzymatic genes involved in ascorbic acid, carotenoid and flavonoid metabolism [22]. The transcriptome sequencing data of cultivar Heinz and the wild relative *Solanum pimpinellifolium* were also downloaded from TFGD (http://ted.bti.cornell.edu/cgi-bin/TFGD/digital/experiment.cgi?ID=D004). In order to exclude false positives, structural genes and transcription factors with an RPKM value ≥5.0 in at least one of the seven stages during fruit development were selected, and transcription factors with correlation coefficient values of ≥0.8 by *t* test (The formula to calculate *t* value was *t* = $\frac{\text{r}\sqrt{n-2}}{\sqrt{1-r^{2}}}$, at *P<0*.*05* and n = 7. |*t*|>*t*_0.05,5_ = 2.571 means significant correlation, so r>0.754 means significant correlation) were considered to have an expression that was significantly correlated with the expression of genes in the various biosynthetic pathways. The co-expression analysis was preformed by “CORREL” function in “EXCEL2003” and confirmed by an in-house Perl scripts and IBM SPSS Statistics software.

### Agroinfiltration

The full length cDNAs of three transcription factors (MYB [Solyc09g010840.1], NAC [Solyc12g013620.1] and ZIF [Solyc06g065440.1]) were amplified from the AC by reverse transcription (RT)-PCR. The PCR products were inserted into the entry vector pDONR221 using the BP enzyme (Invitrogen, USA), and then cloned into the destination vector pMV3 using the Gateway recombination reaction (Invitrogen, USA). The resultant constructs, as well as the pMV3 empty vector (control), were introduced into the *Agrobacterium tumefaciens* strain EHA105 and agroinfiltration of AC fruits was carried out as previously described [27]. For each construct, three fruits from the same position of two independent plants were agroinfiltrated. RNA were isolated from infiltrated fruits and analyzed by qRT-PCR. The agroinfiltration experiments were repeated three times. The sequences of the primer pairs (designed using Primer Premier 3.0) are listed in S1 Table.

## Results

### Changes in ascorbic acid, carotenoids and flavonoids content during tomato fruit development and ripening

**Fig 1:**
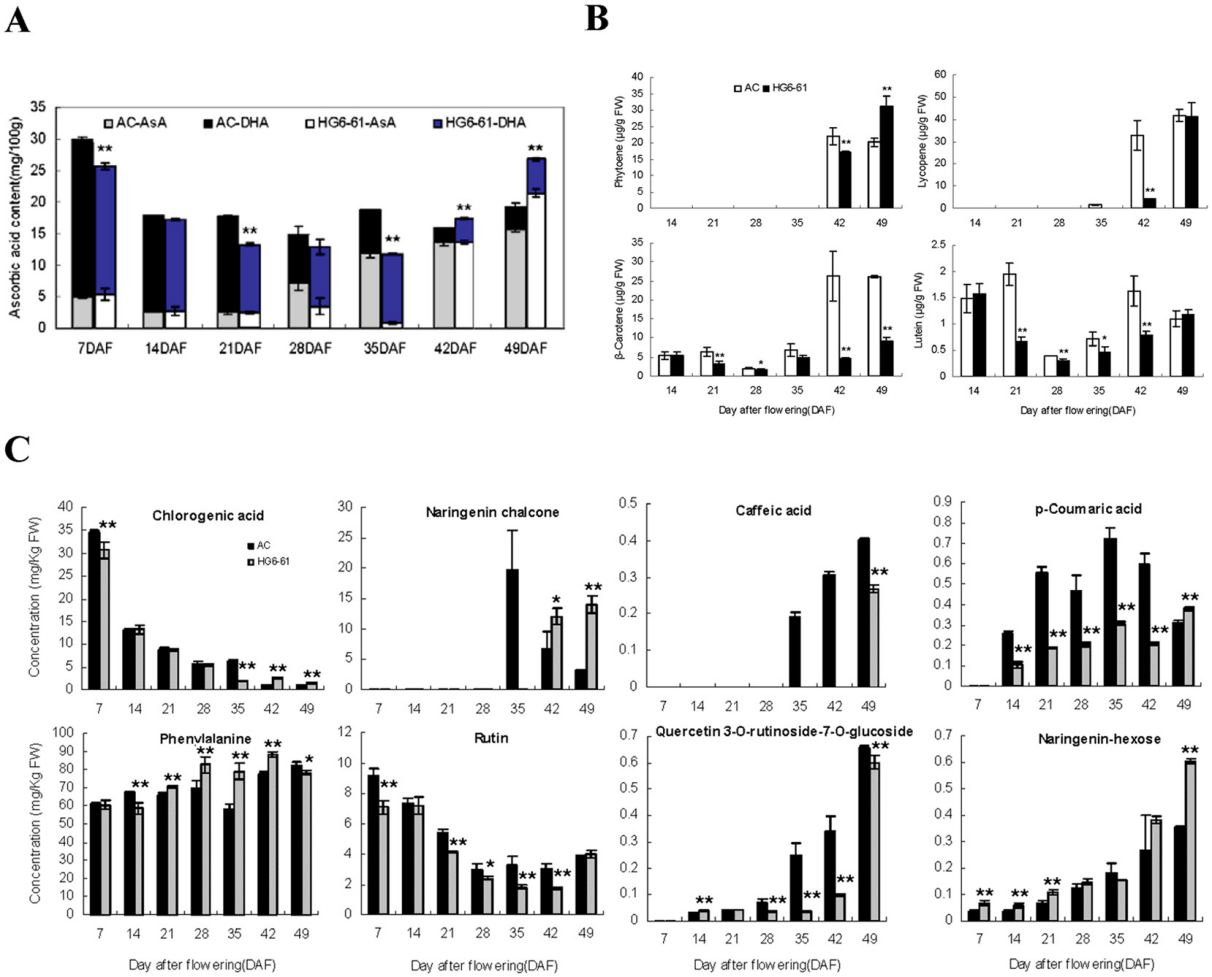
Dynamics of ascorbic acid, carotenoids and flavonoids accumulation during fruit development and ripening. The concentrations of ascorbic acid (A) and carotenoids (B) were determined by HPLC. The flavonoids contents (C) were determined by LC-MS. DHA (gray squares in AC and light blue squares in HG6-61) and AsA (black squares in AC and white squares in HG6-61) means oxidized ascorbate and reduced ascorbate, respectively in (A). Fruits of AC (black squares) and HG6-61(gray squares) at each of seven selected developmental stages were used in the analysis in (B) and (C). Bars represent the standard error (n = 3). DAF, days after flowering.

Metabolism analysis was carried out to monitor the dynamics of fruit ascorbic acid, carotenoids and flavonoids content in two tomato cultivars AC and HG6-61 (Fig 1). Most metabolites showed similar fluctuations in the two cultivars during fruit development, but the accumulation of some metabolites such as lycopene in carotenoids and naringenin chalcone belonging to flavonoids in AC fruits reached to its peak value earlier than in HG6-61 (Fig 1B and 1C). The content of total ascorbic acid showed a high-low-high pattern along fruit development and ripening but higher in HG6-61 than AC at 49 DAF (Fig 1A). The carotenoid accumulation showed a increasing trend in two cultivars but faster in AC than HG6-61 (Fig 1B). For flavonoids, most of the metabolites showed increasing trend along fruit development and ripening except that chlorogenic acid and rutin declined gradually toward maturation (Fig 1C). When comparing the metabolite concentration between two cultivars, most of final metabolite concentration is equal except that difference occurred in ascorbic acid, phytoene, β-carotene, naringenin chalcone, caffeic acid, and naringenin-hexose. The metabolite accumulation difference in the later development stages of two genotypes is possibly due to the different maturation progress.

### Transcriptome profiling of seven tomato fruit developmental stages

We used RNA-seq to profile the transcriptomes of fruit from the tomato cultivars AC and H6-61 at 7 time points: 7, 14, 21, 28, 35, 42 and 49 DAF. The average number of reads produced for each sample was 9.5 million (Table 1), with the number of clean reads per sample ranging from 3.3 to 10.9 million. The number of reads that were mapped to the *S*. *lycopersicum* genome ranged from 1,786,380 to 6,780,667, and the uniquely mapped reads from 1,657,266 to 6,576,631. We found that most of the uniquely mapped reads were mature mRNA or non-coding RNA (ncRNA), and the reads that mapped multiple times were primarily ribosomal RNA (rRNA) or transfer RNA (tRNA). Only uniquely mapped reads were used in subsequent analyses. A read distribution analysis revealed that >70% of the uniquely mapped reads originated from coding sequences (CDS), while the rest were distributed in intergenic regions, 3’UTRs (untranslated regions), introns or 5’UTRs (Table 1). mRNA coverage analysis suggested the set of sequences derived from the RNA-seq analysis covered the complete region of mRNAs, with most reads falling in 20–80 relative position of the mRNA (S1 Fig). RPKM statistics showed that approximately 60% of the genes in each sample had RPKM values < 20, suggesting that most of the genes were expressed at low levels (S2 Fig), likely reflecting the fact that the plant does not express all its genes during its entire life cycle.

**Table 1:** Overview of RNA-seq data from Ailsa Craig (AC) and HG6-61 at 7 fruit developmental stages.

Category		7 DAF	14 DAF	21 DAF	28 DAF	35 DAF	42 DAF	49 DAF
Raw reads	AC	9743349	8621393	5433561	9323506	9368088	7530118	9268675
	HG6-61	10004399	14596765	17479136	8339706	7970371	6674007	8624540
Clean reads	AC	4810773	5538408	3320796	6644534	6543483	4348086	6192442
	HG6-61	6370206	8294104	10917553	6071110	3951893	4293645	5033699
Total mapped reads ^a^	AC	2020456 (42.00%)	2753665 (49.72%)	2209561 (66.54%)	4505071 (67.80%)	4729099 (72.27%)	2122941 (48.82%)	3039356 (49.08%)
	HG6-61	4341791 (68.16%)	4849222 (58.47%)	6780667 (62.11%)	4205811 (69.28%)	1786380 (45.20%)	2384589 (55.54%)	2370331 (47.09%)
Uniquely mapped reads ^b^	AC	1936897 (95.86%)	2660025 (96.60%)	2147706 (97.20%)	4395738 (97.57%)	4523181 (95.65%)	2009721 (94.67%)	2854199 (93.91%)
	HG6-61	4191949 (96.55%)	4695471 (96.83%)	6576631 (96.99%)	4090416 (97.26%)	1657266 (92.77%)	2263693 (94.93%)	2225325 (93.88%)
Multiple mapped reads ^b^	AC	83559 (4.14%)	93640 (3.40%)	61855 (2.80%)	109333 (2.43%)	205918 (4.35%)	113220 (5.33%)	185157 (6.09%)
	HG6-61	149842 (3.45%)	153751 (3.17%)	204036 (3.01%)	115395 (2.74%)	129114 (7.23%)	120896 (5.07%)	145006 (6.12%)
Expressed gene (mapped reads no.>0) ^c^	AC	20084 (57.83%)	20470 (58.95%)	21034 (60.57%)	21427 (61.70%)	20199 (58.17%)	17346 (49.95%)	18251 (52.56%)
	HG6-61	21425 (61.70%)	21836 (62.88%)	22493 (64.77%)	21420 (61.68%)	19427 (55.94%)	18573 (53.48%)	17947 (51.68%)
Expressed gene (mapped reads no.>10) ^c^	AC	14542 (72.41%)	15230 (74.40%)	14990 (71.27%)	16405 (76.56%)	14803 (73.29%)	11776 (67.89%)	13165 (72.13%)
	HG6-61	16479 (76.91%)	16966 (77.70%)	17774 (79.02%)	16340 (76.28%)	12438 (64.02%)	12878 (69.34%)	12699 (70.76%)
CDS ^c^	AC	1523820.46 (78.7%)	2099771.55 (78.9%)	1674726.97 (78.0%)	3453778.09 (78.6%)	3386878.32 (74.9%)	1541112.56 (76.7%)	2150765.24 (75.4%)
	HG6-61	3343468.52 (79.8%)	3645901.76 (77.6%)	5130825.65 (78.0%)	3243123.13(79.3%)	1116584.20 (67.4%)	1738604.77 (76.8%)	1728514.98 (77.7%)
5’UTR ^c^	AC	52263.92 (2.7%)	79649.91 (3.0%)	68780.63 (3.2%)	135079.93 (3.1%)	94655.01 (2.1%)	53667.99 (2.7%)	68782.74 (2.4%)
	HG6-61	132005.56 (3.1%)	151683.56 (3.2%)	227801.97 (3.5%)	145053.75 (3.5%)	39452.79 (2.4%)	62592.43 (2.8%)	63391.43 (2.8%)
Intergenic ^c^	AC	183083.63 (9.5%)	234708.06 (8.8%)	195998.25 (9.1%)	398797.04 (9.1%)	479653.13 (10.6%)	214878.40 (10.7%)	321297.47 (11.3%)
	HG6-61	379218.12 (9.0%)	460551.51 (9.8%)	615875.91 (9.4%)	370704.45 (9.1%)	263880.45 (15.9%)	249794.60 (11.0%)	230839.58 (10.4%)
intron ^c^	AC	67507.83 (3.5%)	108012.24 (4.1%)	94782.38 (4.4%)	195180.62 (4.4%)	158333.53 (3.5%)	71345.48 (3.6%)	112818.95 (4.0%)
	HG6-61	159506.23 (3.8%)	200093.70 (4.3%)	294478.34 (4.5%)	178768.06 (4.4%)	67442.53 (4.1%)	89116.56 (3.9%)	89112.94 (4.0%)
3’UTR ^c^	AC	110221.16 (5.7%)	137883.25 (5.2%)	113417.77 (5.3%)	212902.32 (4.8%)	403661.00 (8.9%)	128716.57 (6.4%)	200534.60 (7.0%)
	HG6-61	177750.57 (4.2%)	237240.48 (5.1%)	307649.12 (4.7%)	152766.61 (3.7%)	169906.02 (10.3%)	123584.64 (5.5%)	113466.07 (5.1%)

^a^ The numbers in brackets indicate the percentages of clean reads.

^b^ The numbers in brackets indicate percentages of total mapped reads.

^c^ The numbers in brackets indicate the percentages of uniquely mapped reads.

### Changes in the transcriptome during fruit development and ripening

**Fig 2:**
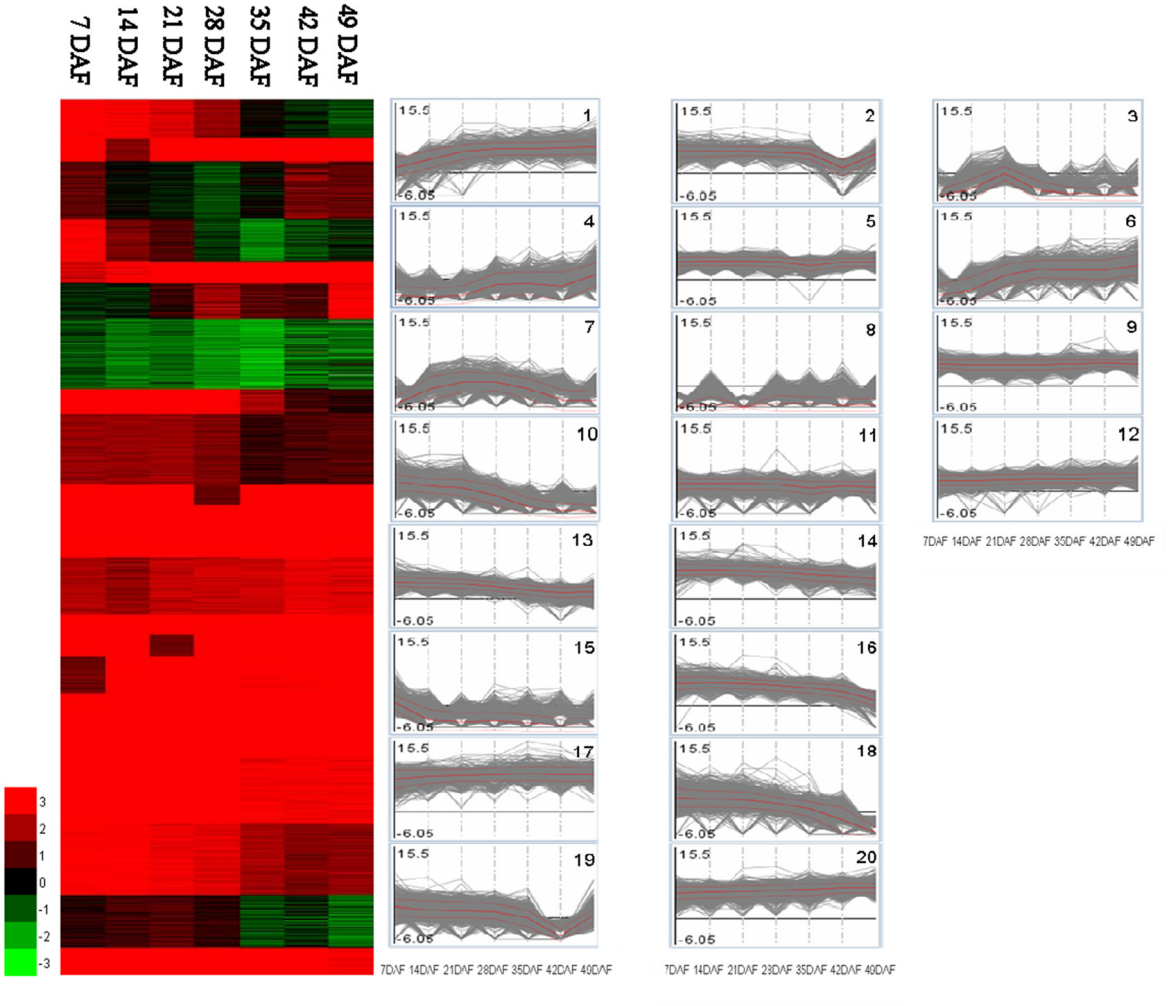
Transcriptome dynamics in Ailsa Craig fruit during development. The log_2_ value of reads per kilobase of a gene per million reads (RPKM) for each gene was used for the K-mean clustering analysis of each of the seven selected developmental stages (7, 14, 21, 28, 35, 42 and 49 days after flowering [DAF]). The 26,397 genes were grouped into 20 expression patterns. The designation is based on the nomenclature of the gene expression pattern.

In this study, we used two tomato cultivars, AC and HG6-61, to reveal the changes in the transcriptome during fruit development and ripening. It was found that approximately 70% of the total numbers of detected genes were expressed at any one fruit developmental stage (Table 1). A total of 26,397 tomato genes were expressed in at least one of the seven sampling points, accounting for 76% of the 34,727 genes in the tomato reference genome, suggesting that the RNA-seq experiment gave a saturated coverage of expression (S2 Table). Among the 26,397 genes detected, 14,758 were expressed in all seven stages and five of these genes were most highly expressed in AC at all stages, with RPKM values >4,000. Two of them, *Solyc05g053070*.*2* and *Solyc05g054090*.*2*, are located on chromosome 5 and encode proteins with unknown function. Two others, located on chromosome 1, encode a pre-mRNA-splicing factor ATP-dependent RNA helicase (*Solyc01g110700*.*2*) and the CCR2 glycine-rich RNA-binding protein (Solyc01g109660.2). The fifth gene (*Solyc11g008510*.*1*) encodes a 60S ribosomal protein. Transcriptome changes during fruit development and ripening were examined using a K-mean cluster analysis of gene expression patterns, which divided the 26,397 genes into 20 groups (Fig 2). Each group exhibited was characterized by a unique expression pattern and the largest group (8) included 3,523 (13.2%) genes, most of which were not expressed at 7 DAF or 21 DAF, but maintained a relative stable expression at 14 DAF and in the last four stages. This group included genes related to cell wall biology, protein modulation and RNA regulation. The second largest group (20) contained 2,763 (10.5%) genes showing a stable high expression throughout all seven stages and the functions of most genes in this group were associated with the ‘protein’ and ‘development’ categories. The expression levels of 2,130 (8.1%) genes in group 19 declined gradually from 7 DAF toward 42 DAF, and then rose at 49 DAF. The transcript abundance of some genes in group 2 showed induction upon ripening from 42 DAF to 49 DAF. A comparison of the expression patterns between AC (Fig 2) and HG6-61 (S3 Fig) showed that all the identified genes could be similarly arranged into the 20 pattern groups.

**Fig 3:**
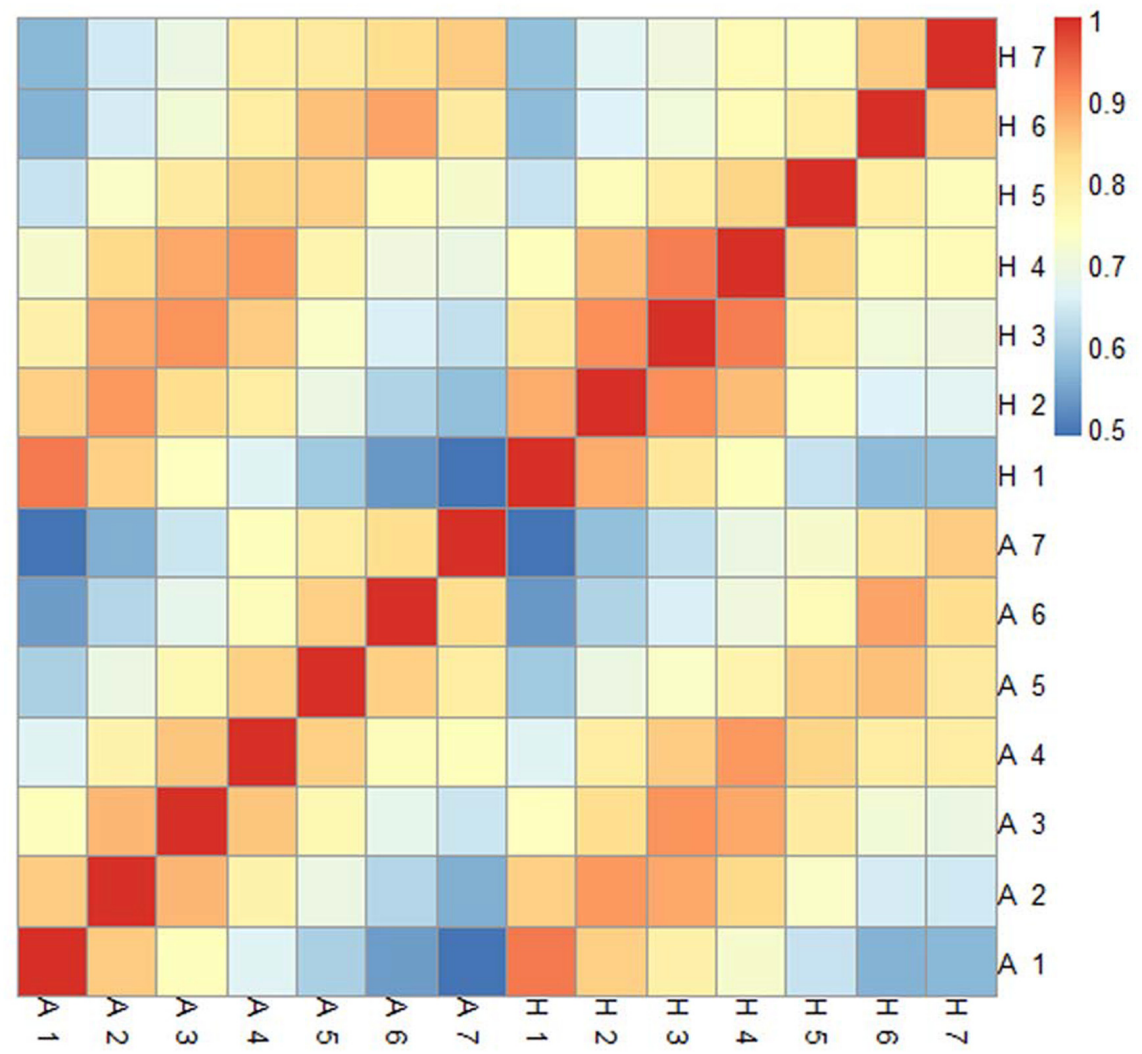
Correlation analysis of gene expression among different developmental stages in two tomato cultivars. Correlation was evaluated according to expression levels of each gene in the different sampling points of Ailsa Craig (A) and HG6-61 (H). The numbers 1 to 7 indicate 7, 14, 21, 28, 35, 42 and 49 DAF, respectively.

A correlation analysis using the 14 sampling points from the two tomato cultivars also showed that AC and HG6-61 had similar transcription patterns at each development stage (Fig 3), so there was a strong resemblance in terms of gene expression between AC and HG6-61 during fruit development. According to the correlation coefficient analysis, we observed that the number of differentially expressed genes increased through fruit development, with the correlation coefficient decreasing toward ripening for both cultivars. The number of differentially expressed genes peaked at 42 DAF and 47 DAF in HG6-61 and AC, respectively (S4 Fig). While the gene expression patterns in the two cultivars were similar throughout fruit development and ripening, but the correlation coefficient showed a gradual decrease from 0.921 to 0.823 during ripening suggesting different ripening process in AC and HG6-61, consistent with metabolism analysis.

**Fig 4:**
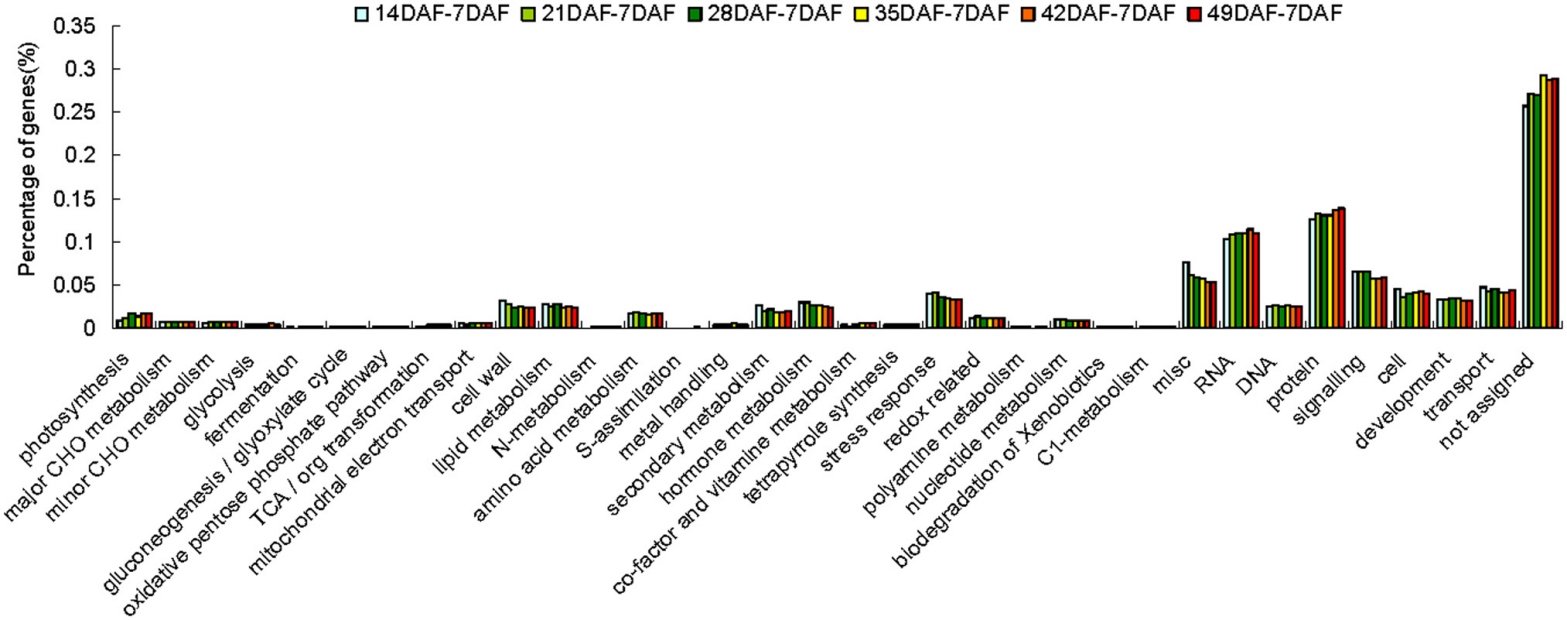
Functional categorization of differentially expressed genes during tomato fruit development in Ailsa Craig. The differences between 14 and 7 DAF are indicated by light green squares (∎). The differences between 21 and 7 DAF are indicated by green squares (∎). The differences between 28 and 7 DAF are indicated by dark green squares (∎), The differences between 35 and 7 DAF are indicated by yellow squares (∎), The differences between 42 and 7 DAF are indicated by orange squares (∎), The differences between 49 and 7 DAF are indicated by red squares (∎). Percentages are calculated based on the proportion of the number of genes in each set.

**Fig 5:**
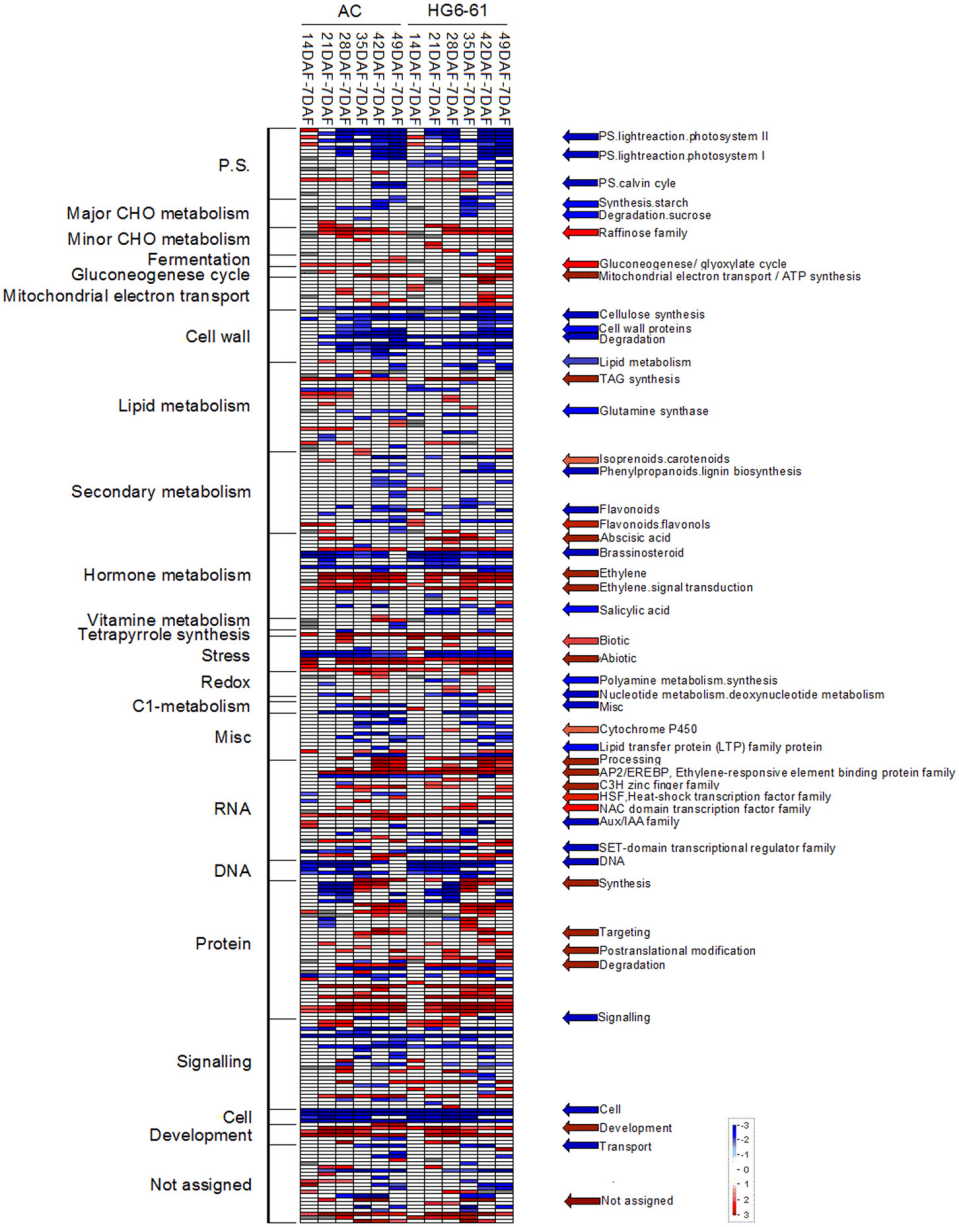
Main pathways expressed during fruit development in the two genotypes (AC and HG6-61). Gene expression data are presented as log_2_ fold change values compared to the first sampling point (7 DAF) within each genotype. The data were subjected to a Wilcoxon test in PageMan [46], and the results are displayed as a false-color code. Bins colored in red correspond to genes that were significantly up-regulated and bins colored in blue correspond to genes that were significantly down-regulated.

Of the 26,397 genes, 3,531, 5,576, 7,616, 8,645, 8 968 and 9,878 were differently expressed at 14 DAF, 21 DAF, 28 DAF, 35 DAF, 42 DAF and 49 DAF, respectively, compared to 7 DAF in AC fruit. In HG6-61 fruit, 3,677, 5,685, 7,391, 8,050, 8,946 and 8,881 genes were differentially expressed at these same stages compared to 7 DAF (S5 Fig). To identify significantly altered biological processes during fruit development and ripening based on these data, the differentially expressed genes from the seven stages of both cultivars were subjected to a GO term enrichment analysis. The differentially expressed genes were divided into 34 groups including “RNA regulation”, “cell wall”, “secondary metabolism”, “hormone metabolism”, “stress”, “lipid metabolism”, “protein”, “signaling”, “development” and “transport” (Fig 4) and this analysis also indicated that the two genotypes had similar transcript expression patterns (Fig 5). Some groups, such as “photosynthetic light reactions”, and “cell wall”, contained genes that showed continuous down-regulation during fruit development and ripening, while other groups were comprised of genes that showed the opposite pattern; specifically “development”, “ethylene response/ signaling pathways”, “ABA response” and “hormone metabolism”. Transcript abundance of genes involved in the “micro RNA, natural antisense” category did not change substantially during fruit development, while the expression of several genes changed dramatically during fruit development and ripening in AC (Table 2).

**Table 2:** List of genes involved in major processes associated with tomato fruit development.

Go term	Gene identifier	Gene description	14 DAF vs 7 DAF Fold change	21 DAF vs 14 DAF Fold change	28 DAF vs 21 DAF Fold change	35 DAF vs 28 DAF Fold change	42 DAF vs 35 DAF Fold change	49 DAF vs 42 DAF Fold change
**RNA regulation**	Solyc01g091000.2	Zinc-binding protein	0.35	0.3	0.16	-	-	-
	Solyc06g069220.1	Aspartl protease family protein	1.92	3.62	1.86	0.42	0.7	0.79
	Solyc12g087940.1	Chloroplast nucleoid DNA-binding protein	0.01	1.02	0.29	0.65	-	-
	Solyc08g077940.1	Histone methyltransferase	0.4	0.15	0.17	-	-	-
	Solyc05g014710.2	Remorin family protein	0.2	0.48	0.28	0.06	-	0.28/0
	Solyc03g113550.2	Basic helix-loop-helix (bHLH) family protein	0.29	1.35	0.2	0.56	2	-
	Solyc11g020670.1	TCP family transcription factor	2.36	1.85	2.25	1.58	1.41	0.76
	Solyc03g026020.2	HSFB2A DNA binding transcription factor	4.1	1.69	1.4	1.88	1.17	1.68
	Solyc05g007180.2	ATHB13 DNA binding transcription factor	0.42	0.39	0.31	0.23	0.09	3.25
	Solyc07g006570.2	Ribonuclease T2 family protein	0.12	0.24	0.34	1.16	0.36	0.29
**secondary metabolism**	Solyc10g052490.1	Isoflavone reductase	0.21	0.37	0.46	4.5	-	-
	Solyc07g054920.1	2OG-Fe (II) oxygenase family protein	5.75	2.92	0.1	0	-	-
	Solyc09g059170.1	Glycosyltransferase family protein	0.45	0.18	0.19	12.2	0.06	-
	Solyc07g043500.1	UDP-glucoronosyl transferase family protein	0.05	0.25	0.23	0.24	0.58	1.41
	Solyc03g114800.1	Desulfoglucosinolate sulfotransferase	0.29	0.44	0.11	0.56	-	-
	Solyc02g093270.2	Caffeoyl-CoA 3-O-methyltransferase	12.29/0	0.5	6.7	0.12	1.31	7.09
	Solyc11g010960.1	Aryl-alcohol dehydrogenase	1.17	0.64	0.28	0.06	-	-
	Solyc08g080170.2	Acetyl-CoA C-acetyltransferase	0.18	0.53	0.79	0.16	2.16	1.05
	Solyc01g088400.2	Octadecanal decarbonylase	0.14	0.3	0.26	434.39	0.74	0.61
	Solyc11g012260.1	Acyltransferase	0.18	4.32	3.02	0.49	1.02	2.1
**hormone metabolism**	Solyc06g007910.2	Gibberellin-regulated GASA family protein	1.15	0.5	0.12	0.25	-	-
	Solyc03g006880.2	Gibberellin 20-oxidase	1.77	0.02	1.95	0	-	-
	Solyc02g064690.2	N-acetyltransferase	1.96/0	4.94	1.25	2.16	0.62	0.09
	Solyc02g077370.1	ERF1 transcription factor	0.77/0	14.81	4.01	0.23	1.08	17.18
	Solyc02g036350.2	1-aminocyclopropane-1-carboxylate oxidase	11.88	4.77	1.16	2.63	0.01	7.22
	Solyc07g049530.2	1-aminocyclopropane-1-carboxylate oxidase	3.52	2.58	22.94	0.38	0.55	4.77
	Solyc02g079190.2	Transport inhibitor response 1	1.83	0.64	0.63	0.57	1.45	0.84
	Solyc05g008060.2	Auxin:hydrogen symporter	1.12	0.4	1.25	0.39	0.06	10.34
	Solyc10g083170.1	Alcohol dehydrogenase	0.01	4.93	0.12	42	2.25	0.02
	Solyc07g056570.1	9-cis-epoxycarotenoid dioxygenase	11.79	4.73	1.68	0.52	0.3	0.4
**cell wall**	Solyc12g008530.1	Pectinesterase family protein	0.32	0.44	0.89	0.49	-	1.22/0
	Solyc03g071570.2	Pectate lyase family protein	0.55	0.56	0.41	0.24	0.13	-
	Solyc05g005560.2	BURP domain-containing protein	5.29	0.94	0.37	0.05	0.11	0.24
	Solyc07g065090.1	Polygalacturonase inhibiting protein 1	0.11	0.43	0.24	1.07	-	12.11/0
	Solyc01g008710.2	(1,4)-beta-mannan endohydrolase	0.26/0	-	0.47/0	958.68	0.01	0.04
	Solyc07g043390.2	Cellulose synthase	0.01	1.11	0.53	0.07	2.26	0.35
	Solyc02g088690.2	UDP-glucose 6-dehydrogenase	0.87	1.04	0.93	0.6	1.14	2.5
	Solyc06g051800.2	Expansin A4	1.1	2.45	23.3	10.33	0.19	0.07
	Solyc07g052980.2	Xyloglucan endotransglucosylase/hydrolase 9	0.08	0.71	0.1	0.49	-	-
	Solyc10g083670.1	mannan synthase	0.99	0.93	0.05	1.54	0.82	-
**stress**	Solyc00g060810.2	MLP-LIKE PROTEIN 43	-	-	26.06/0	26.11	1.85	0.25
	Solyc02g089250.2	Allergen and extensin family protein	0.2	0.04	0.45	1.64	0.06	-
	Solyc02g062320.2	RD22; nutrient reservoir	53.98	0.85	0.03	-	-	-
	Solyc03g098740.1	Trypsin and protease inhibitor family protein	1.22	0.62	43.91	1.72	0.86	20.76
	Solyc07g006380.2	Low-molecular-weight cysteine-rich 75	0.02	0.05	-	-	-	-
	Solyc07g053020.1	Disease resistance protein	1.78	0.24	1.15	0.5	1.12	0.5
	Solyc07g006700.1	Pathogenesis-related protein	0.11	0.04	-	0.75/0	-	-
	Solyc08g080630.2	Protease inhibitor	0.62/0	1.24	2.79	3.57	1.43	17.74
	Solyc10g081980.1	NHL3	6.61	14.56	0.76	0.91	2.54	1.26
	Solyc10g085890.1	UDP-glycosyltransferase	16.25	1.84	1.81	2.19	0.64	0.09

For each gene, the number given at each stage indicates the fold change in expression level compared with the level at the previous stage:—indicates that no expression was detected in that stage; 0, zero detectable expression. For example, 12.29/0 indicates that expression was not detected at the earlier sampling stage, but the transcript expression value is 12.29 in the indicated stage.

Two transcription factors in the “RNA regulation” category (*Solyc01g091000*.*2*, *Solyc12g087940*.*1*) showed a decrease in expression throughout fruit development and ripening, while another transcription factor (*HSFB2A*, *Solyc03g026020*.*2*) showed an increase in expression during fruit maturation. In contrast, the expression of a homolog of the *A*. *thaliana* ATHB13 DNA binding transcription factor (*Solyc05g007180*.*2*) declined during early fruit development but increased sharply at the mature stage.

The expression of several genes involved in secondary metabolism (e.g. flavonoid and phenylpropanoid biosynthesis) showed notable changes during fruit development. For example, the transcript abundance of three flavonoid related genes (*Solyc10g052490*.*1*, *Solyc09g059170*.*1*, *Solyc07g043500*.*1*) decreased during fruit development and ripening, while a gene encoding a 2OG-Fe(II) oxygenase family protein (*Solyc07g054920*.*1*), which is involved in flavonoid metabolism (http://solgenomics.net/), was up-regulated at the early stages of fruit development and then declined sharply to undetectable levels at the final ripening stage. The expression of a gene encoding the caffeoyl-CoA 3-O-methyltransferase (*Solyc02g093270*.*2*), which is associated with phenylpropanoid metabolism (http://solgenomics.net/), similarly showed an ‘up-down-up pattern’ and peaked at 28 DAF and 49 DAF.

In the context of hormone biology, two genes related to gibberellin (GA) synthesis and signaling (*Solyc06g007910*, *Solyc03g006880*.*2*) showed decreasing expression during fruit development and ripening, while genes related to ethylene biosynthesis (*Solyc02g036350*.*2*, *Solyc07g049530*.*2*) and signaling (*Solyc02g077370*.*1*) exhibited increased expression during the later stages of fruit maturation. However, an ethylene response factor 1 gene (*Solyc12g010520*.*1*) showed stable expression throughout fruit development.

Some of the differentially expressed genes are annotated as being associated with cell wall metabolism and fruit softening. For example a gene encoding a pectinesterase (*Solyc12g008530*.*1*) showed a decreasing expression pattern during early development but was up-regulated in mature fruits, which is consistent with the well characterized phenomenon of pectin modification in ripening fruit [25]. In contrast, genes encoding a pectate lyase (*Solyc03g071570*.*2*) and a xyloglucan endotransglucosylase hydrolase (*Solyc07g052980*.*2*) showed a pattern of deceasing expression during fruit ripening. Other patterns were also observed: the transcript levels of an expansin (*Solyc06g051800*.*2*) increased during the early stages of fruit development, peaked at 35 DAF and then gradually declined, while an endo-1,4-β-mannanase gene (*Solyc01g008710*.*2*) showed high expression at 42 DAF but was barely detectable at other stages.

### Transcription factors associated with enzymes involved in ascorbic acid, carotenoid and flavonoid metabolism

The flavonoid, carotenoid and ascorbic acid biosynthetic pathways have been well characterized in terms of the constituent enzyme encoding genes [35–37]. However, the regulators or transcription factors that modulate the expression of these genes remain largely unknown and so we sought to identify some candidates based on the transcriptome analysis. In total 46, 18, and 14 structural genes putatively involved in the ascorbic acid, carotenoid and flavonoid metabolic pathways, respectively, were selected (S3 Table). Based on the transcriptome sequencing data of AC and HG6-61, a transcription abundance correlation analysis was carried out between selected structural genes from the three pathways and 823 annotated tomato transcription factors (S4 Table) and the transcription factors whose expression levels were highly correlated with those of the structural genes were identified (Table 3). Moreover, some metabolic related transcription factors were validated by two sequenced varieties cultivar Heinz and the wild relative *Solanum pimpinellifolium* (S5 Table).

**Table 3:** Correlation analysis of structural genes (SGs) involved in ascorbic acid, carotenoid and flavonoid metabolism and transcription factors (TFs).

TF	Flavonoids ^a^		TF	Ascorbic acid ^a^		TF	Carotenoids ^a^	
	AC	HG6-61		AC	HG6-61		AC	HG6-61
bHLH (Solyc05g006650.2)	7(7)	4(4)	Myb (Solyc09g010840.1)	25(20)	17(17)	CCT domain (Solyc01g106030.2)	9(4)	6(2)
Myb 12 (Solyc01g079620.2)	6(6)	7(7)	AUX/IAA (Solyc03g120500.2)	24(20)	15(15)	MADS-box (Solyc02g084630.2)	9(5)	9(8)
BZR1 (Solyc02g071990.2)	6(0)	2(0)	NAC (Solyc12g013620.1)	24(3)	14(0)	CCT domain (Solyc03g083400.2)	9(6)	7(6)
HSF (Solyc03g026020.2)	5(1)	4(0)	AUX/IAA (Solyc09g090910.1)	23(19)	15(15)	CCT domain (Solyc04g049670.2)	9(6)	8(7)
ZIF CCCH-type (Solyc01g087030.2)	5(1)	3(0)	Dof (Solyc08g008500.2)	23(20)	9(9)	bHLH (Solyc01g096050.2)	9(3)	8(0)
HSF A3 (Solyc03g006000.2)	5(1)	3(1)	ZIF C2H2-type (Solyc06g065440.1)	23(18)	7(7)	LOB domain (Solyc03g119530.2)	9(3)	7(2)
bHLH (Solyc09g083220.2)	5(1)	3(1)	SET(Solyc03g044380.2)	22(19)	15(15)	SBP-box (Solyc05g053240.2)	9(6)	6(5)
Myb (Solyc02g088190.2)	4(4)	7(7)	CCT domain (Solyc03g083400.2)	22(17)	15(15)	YABBY (Solyc11g071810.1)	9(6)	5(3)
LIM (Solyc06g071310.2)	4(4)	6(6)	TCP (Solyc08g048390.1)	22(17)	14(14)	ARF4 (Solyc11g069190.1)	8(6)	8(7)
WRC1 (Solyc07g041640.2)	4(4)	4(4)	MADS-box (Solyc02g084630.2)	22(18)	14(14)	SET (Solyc03g044380.2)	7(3)	8(8)
Myb-like (Solyc08g077230.2)	4(4)	4(4)	LIM (Solyc06g071310.2)	21(17)	18(18)	AUX/IAA (Solyc03g120500.2)	8(6)	8(6)
TCP (Solyc06g065190.1)	3(3)	6(6)	SBP-box (Solyc03g114850.2)	21(18)	17(15)	AUX/IAA (Solyc01g097290.2)	8(6)	8(7)
Myb-like (Solyc12g017370.1)	3(3)	6(6)	SET (Solyc01g006220.2)	21(18)	14(14)	Unknow (Solyc01g096470.2)	8(6)	7(5)
ZIF CCCH-type (Solyc01g008600.2)	3(3)	5(5)	Myb (Solyc03g112390.2)	21(18)	13(13)	NAC (Solyc12g013620.1)	8(2)	8(1)
bHLH (Solyc06g083170.2)	3(3)	5(5)	ZIF CCCH-type (Solyc01g087030.2)	21(5)	13(0)	TF B3 (Solyc06g073980.2)	9(7)	7(7)
SBP-box (Solyc10g078700.1)	3(3)	5(5)	SBP-box (Solyc05g053240.2)	20(17)	16(14)	TF B3 (Solyc01g108930.2)	8(6)	6(5)
ZIF C2H2-type (Solyc09g007550.2)	2(2)	6(6)	CCT domain (Solyc04g049670.2)	20(15)	16(16)	GRAS (Solyc02g085340.1)	8(6)	6(5)
GATA TF 25 (Solyc04g076530.2)	2(1)	7(0)	Unkown (Solyc01g096470.2)	20(17)	14(14)	ZIFCCCH-type (Solyc09g074640.2)	7(5)	6(5)
Myb (Solyc04g078420.1)	2(1)	6(0)	Dof (Solyc08g082910.1)	20(15)	11(9)	Dof (Solyc08g082910.1)	8(6)	6(4)
ERF4 (Solyc07g053740.1)	2(0)	6(0)	HSF A3 (Solyc03g006000.2)	19(5)	16(0)	SBP-box (Solyc03g114850.2)	8(5)	7(5)
			bHLH (Solyc01g096050.2)	19(2)	16(0)	ZIF CCCH-type (Solyc01g087030.2)	7(3)	7(1)
			GRAS (Solyc11g011260.1)	19(17)	16(16)	bHLH (Solyc09g083220.2)	8(2)	7(1)
			SPL3 (Solyc10g009080.2)	19(16)	16(16)	HD-ZIP (Solyc06g060830.2)	7(6)	9(7)
			AUX/IAA (Solyc12g007230.1)	19(16)	16(16)	TF B3 (Solyc02g065350.2)	7(5)	9(7)
			TF B3 (Solyc06g073980.2)	19(17)	15(15)	HSF (Solyc02g090820.2)	7(1)	10(2)
			ORCS 1 (Solyc03g006420.2)	18(16)	17(15)	Myb (Solyc09g010840.1)	7(5)	9(8)
			AUX/IAA (Solyc01g097290.2)	18(16)	15(15)	GRAS (Solyc11g011260.1)	7(5)	9(8)
			ARF8 (Solyc02g037530.2)	18(16)	13(13)	AUX/IAA (Solyc12g007230.1)	7(5)	9(8)
			Myb (Solyc02g067340.2)	17(14)	15(15)	bHLH51(Solyc06g051260.2)	6(5)	7(6)
			ATHB13 (Solyc05g007180.2)	17(16)	14(14)	Myb (Solyc03g112390.2)	7(5)	7(6)
			TCP (Solyc06g065190.1)	16(14)	15(15)	HSF A3 (Solyc03g006000.2)	6(3)	8(2)
			ZIF CCCH-type (Solyc01g008600.2)	15(15)	16(16)	ARF6 (Solyc00g196060.2)	6(4)	7(6)
			GRAS (Solyc08g078800.1)	15(15)	16(16)	TCP (Solyc08g048390.1)	7(6)	5(5)
			NF-YC1 (Solyc06g072040.1)	15(13)	16(16)	SBP-box (Solyc10g078700.1)	8(5)	7(7)
						ZIF C2H2-type (Solyc08g063040.2)	6(1)	5(2)
						CCT domain (Solyc03g119540.2)	5(4)	10(8)
						Myb (Solyc02g067340.2)	6(4)	7(7)

^a^ the number indicates the TFs correlated with SGs, while the number in brackets denotes the TFs positively associated with SGs.

In the ascorbic acid metabolic pathway, the expression of a MYB gene (*Solyc12g088320*.*1*) showed a high correlation with the transcript levels of many of the selected structural genes (25 and 17 of the 46 targeted genes from AC and HG6-61, respectively). In total, 34 transcription factors showed a high correlation with at least 15 structural enzyme encoding genes in AC, and most of them showed a positive correlation with the expression of structural genes, including AUX/IAA (*Solyc03g120500*.*2*, *Solyc09g090910*.*1*), Dof (*Solyc08g008500*.*2*, *Solyc08g082910*.*1*) and LIM (*Solyc06g071310*.*2*). The expression of only four transcription factors, a NAC protein (*Solyc12g013620*.*1*), a CCCH-type zinc finger protein (*Solyc01g087030*.*2*), a HSF A3 protein (*Solyc03g006000*.*2*) and a bHLH protein (Solyc01g096050.2) was negatively correlated with that structural genes involved in the ascorbic acid metabolic pathway. Interestingly, GPI-1, PMI-3, PMM-1, GME-1, GME2, GalDH, GalUR-2, GalUR-3, GalUR-6, AO-2, APX-1, APX-6, APX-7 and MDHAR-3 showed a high positive correlation with most of the 34 transcription factors, while GMP-2, APX-3, APX-10 and DHAR-5 showed a strong negative correlation with many of the 34 transcription factors (S4 Table).

Twenty transcription factors were associated with the flavonoid metabolic pathway, of which 12 had a positive correlation and 8 a negative association (Table 3). MYB12 (*Solyc01g079620*.*2*), which showed a highly positive correlation with many of the selected structural genes (6 and 7 of the 14 selected genes from AC and HG6-61, respectively), is considered to be an important regulator of flavonoid biosynthesis in pink tomato fruit [22]. The expression of other transcription factors, such as bHLH proteins (*Solyc05g006650*.*2*, *Solyc09g083220*.*2* and *Solyc06g083170*.*2*), MYB proteins (*Solyc02g088190*.*2*, *Solyc04g078420*.*1*) and MYB-like proteins (*Solyc08g077230*.*2*, *Solyc12g017370*.*1*) was highly correlated with many of the selected structural genes in the flavonoid pathway.

A total of 37 transcription factors showed a significant correlation with at least 5 structural genes from the carotenoid metabolic pathway in both AC and HG6-61. A MADS-box gene (*Solyc02g084630*.*2*) showed a high correlation with structural genes involved in the carotenoid pathway (8 of 18 selected genes from AC and HG6-61, respectively) and four CCT domain transcription factors (*Solyc01g106030*.*2*, *Solyc03g083400*.*2*, *Solyc03g119540*.*2*, and *Solyc04g049670*.*2*) were positively correlated with the expression of genes in the carotenoid pathway. Other transcription factors, such as AUX/IAA, bHLH, MYB, SBP-box, transcriptional factor B3 and zinc finger proteins also showed a high correlation with carotenoid metabolism, suggesting a complex underlying regulatory network (S4 and S5 Tables).

Furthermore, a correlation analysis was carried out between metabolites accumulation and transcription abundance of structural genes or related transcription factors (S6 Table). Most structural genes and transcription factors involved in ascorbic acid metabolic pathway were significantly positively correlated with oxidized ascorbate (DHA) accumulation, but few were significantly correlated with total ascorbate abundance. The transcription factors showed a higher percentage of correlation with ascorbic acid than structural genes. In flavonoid metabolic pathway, the content of chlorogenic acid and rutin exhibited positive correlation with most structural and regulatory genes, but only two structural genes (*Solyc03g117870* and *Solyc05g010320*) and one transcription factor (*Solyc03g006000*) significantly correlated with naringenin chalcone. It was surprising that the expression level of most structural genes and related transcription factors in carotenoid metabolic pathway didn’t show significant correlation with carotenoids content, including lutein, phytoene, β-Carotene or lycopene.

### Verification of gene expression related to ascorbic acid, carotenoids and flavonoid metabolism

**Fig 6:**
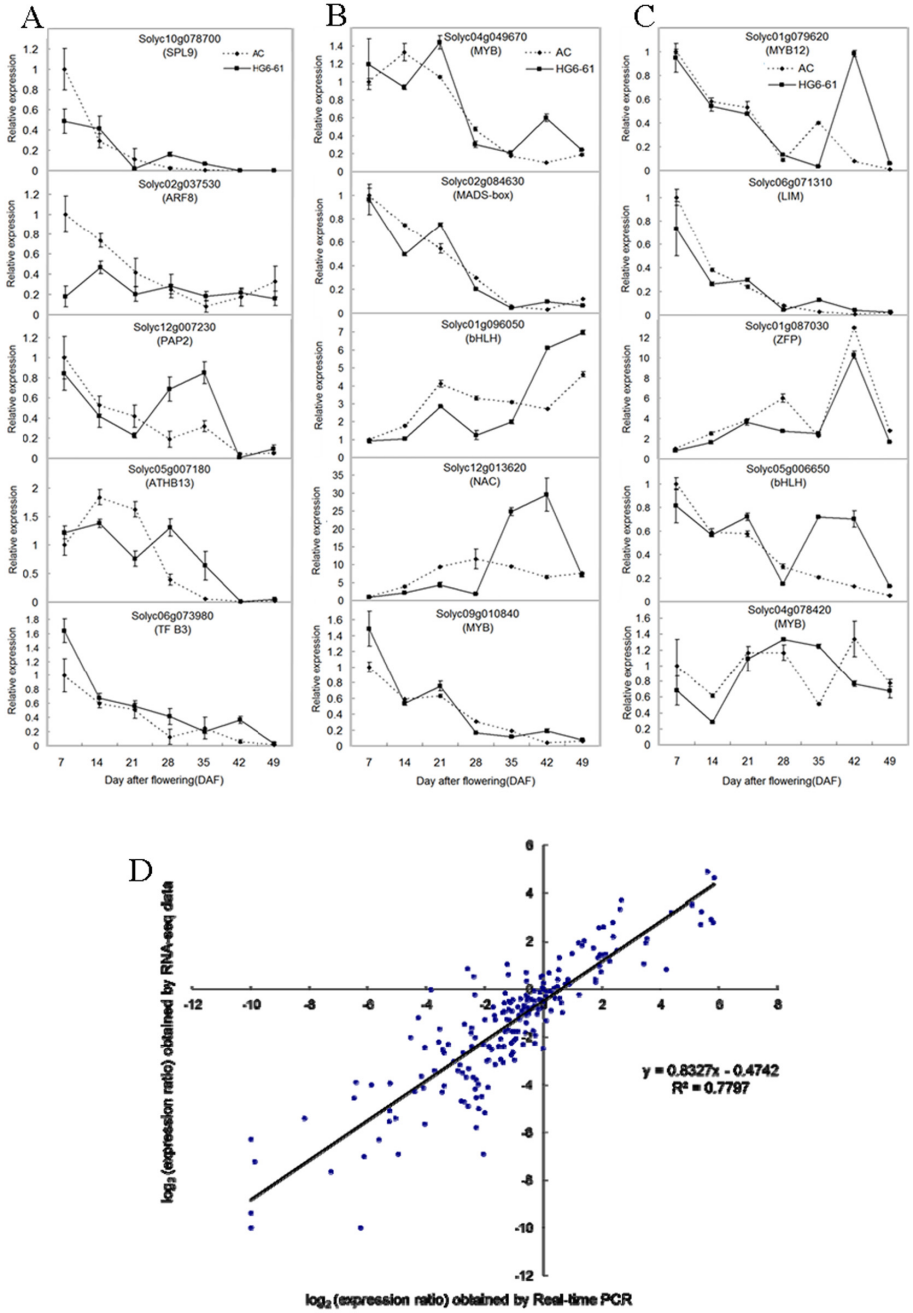
Real-time PCR analyses. Transcript levels of 15 transcription factors, of which 5 are potentially associated with the ascorbic acid pathway (A), 5 are potentially associated with the carotenoids pathway (B) and the last 5 are potentially associated with the flavonoids pathway (C) in Ailsa Craig (broken line) and HG6-61 (solid line). Correlation analysis of the gene expression ratios obtained from the RNA-seq data and the qRT-PCR analysis is presented in D. Results shown represent mean values (±SE) from three independent experiments.

qRT-PCR was employed to verify the RNA-seq based transcription profiles. RNA was extracted from fruits at the same seven developmental stages and cDNA was synthesized as a template for qRT-PCR. The expression of 15 transcription factors putatively related to ascorbic acid, carotenoid and flavonoid biosynthesis was assessed by qRT-PCR (Fig 6A–6C) and correlation analysis revealed a high degree of consistency between transcript abundance determined by qRT-PCR or RNA-seq (Fig 6D).

### Agroinfiltration verification of the association between selected transcription factors and genes encoding enzymes involved in ascorbic acid metabolism

**Fig 7:**
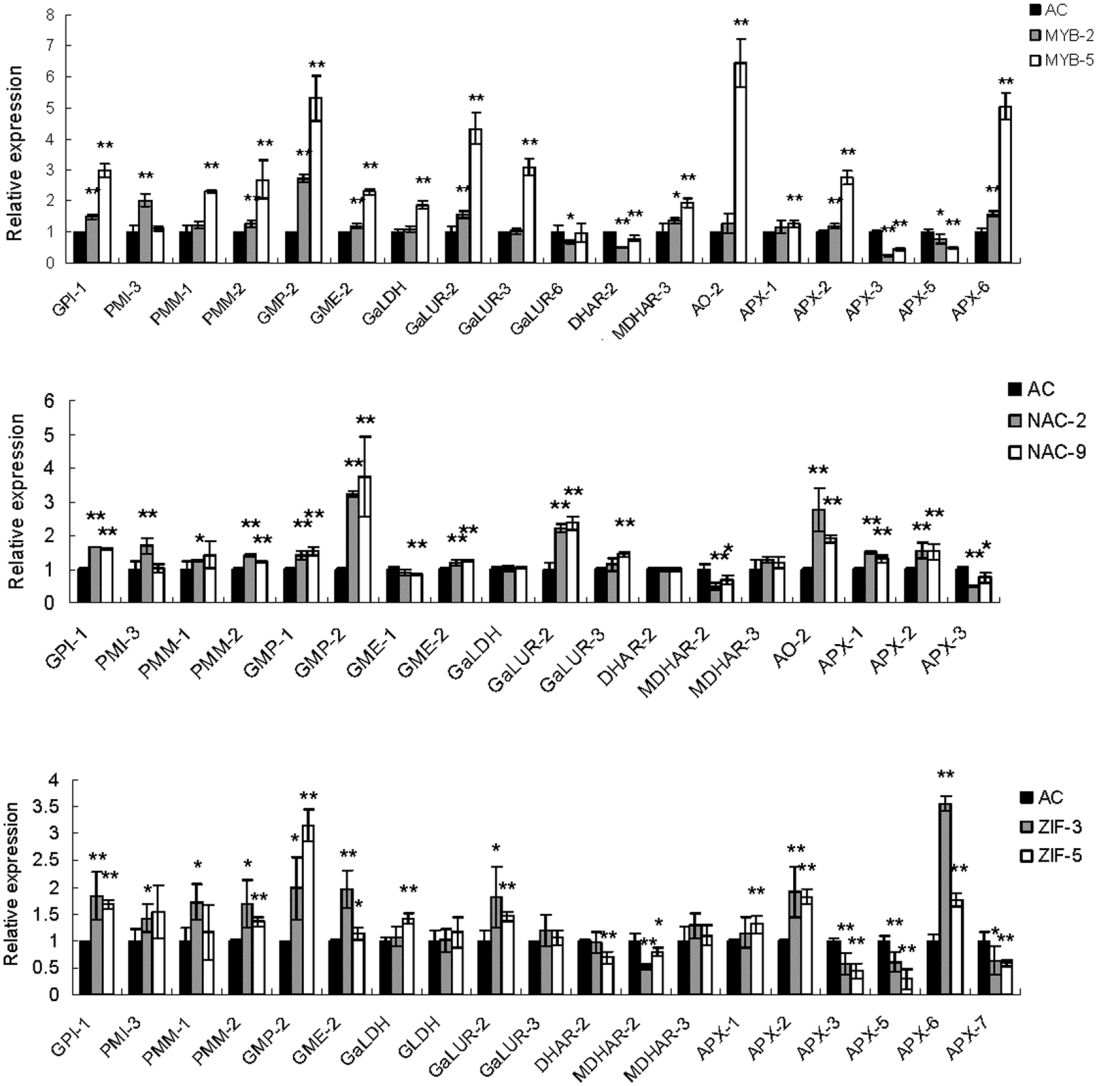
Differential expression of structural genes involved in the ascorbic acid metabolic pathway regulated by transiently expressed transcription factors. The Figure shows transcript levels of different structural genes involved in the ascorbic acid biosynthetic pathway. MYB (*Solyc09g010840*.*1*), NAC (*Solyc12g013620*.*1*) and ZIF (*Solyc06g065440*.*1*) were used for agroinfiltration and AC means agroinfiltrated with empty vector. For each transcription factor, two independent lines were selected Results shown represent mean values (±SE) from three independent experiments. Asterisks indicate significant differences as determined by Student’s t-test (*P<0.*05*; ** *P<0*.*01*).

To further validate the transcriptional association between the transcription factors and structural genes presented in Table 3, we performed agroinfiltration to verify the putative regulatory activity of the selected transcription factors on genes associated with ascorbic acid biosynthesis. Specifically, we selected three representative transcription factors: a MYB protein (*Solyc09g010840*.*1*), a NAC protein (*Solyc12g013620*.*1*) and a ZIF protein (*Solyc06g065440*.*1*) (S6 Fig). After agroinfiltration, the relative expression abundance of the corresponding genes increased at least three fold (MYB-2) and in one instance more than 30 fold (NAC-9). The expression levels of the selected structural genes involved in ascorbic acid metabolism was then assessed (Fig 7). In the MYB over-expressing fruit, most of the structural genes (13 of 18) were up-regulated, including GMP2, GalUR, AO2, and APX6. Only 5 of the genes were either down-regulated (3 of 5) or show no change in expression (2 of 5). A similar expression profile was seen in the NAC and ZIF over-expressing fruit (Fig 7). Furthermore, we found a quantitative relationship between the degree of over-expression of the transgene and the expression of the associated structural genes, further suggesting a correlation between expression of each of the transcription factors and the ascorbic acid biosynthesis genes (Fig 7).

## Discussion

Fruit development has been extensively investigated with regards to ethylene synthesis, signal transduction and cell wall-modifying proteins [25, 38–42], and studies of non-ripening tomato mutants have provided insights into the signal transduction networks that govern ethylene synthesis and production, perception and signaling, as well as other aspects of ripening related metabolism [5]. However, the transcriptional profiles related to fruit nutritional quality have been less studied. Here, two cultivated tomato genotypes, AC and HG6-61, were analyzed by RNA-seq to characterize the genome-wide transcriptome dynamics and differential expression of genes during fruit development and ripening. A total of 26,397 tomato genes were detected in the present study, providing a more detailed overview of gene expression than obtained previously using microarrays [25]. Furthermore, the parallel analysis of two different cultivated tomato varieties, Ailsa Craig, an English heritage variety, and HG6-61, an elite tomato inbred line from China, allowed as assessment of conserved and divergent patterns of gene expression. From this we concluded that the gene expression profiles of the two genotypes were highly similar during fruit development and ripening (Figs 3 and 5) and this high concurrence between two genotypes can prove our transcriptional data is reliable in the absence of replicate. Moreover, given that the expression profiles of genes determined by the RNA-seq evaluation and further validated by q-PCR were extremely similar (Fig 6), we are confident that our RNA-seq data set will be broadly useful for studies of fruit transcriptome dynamics.

Many studies of the tomato fruit transcriptome to date have broadly addressed hormone-mediated fruit ripening, rather than targeting the development of molecular basis of the underlying quality traits [5, 43]. Transcriptome analyses of ripening related tomato mutants, such as *ripening-inhibitor* (*rin*) [25], *non-ripening* (*nor*) and *never-ripe* (*Nr*) [5] have helped dissect processes associated with ethylene-mediated fruit ripening. This late stage of fruit development is accompanied by the accumulation of carotenoids, flavonoids and ascorbic acid, all of which are beneficial to human health. The identity of the factors that regulate the accumulation of these compounds is therefore of great commercial interest. A study of the transcriptomes of orange(*Citrus sinensis*) fruit and its red fleshed mutant showed the differential expression of genes involved in carotenoid metabolism; however, associated transcription factors were not reported [44].

A key hypothesis in this study was that an investigation of the structural genes that contribute to the formation of nutritionally valuable compounds, such as ascorbic acid, carotenoids and flavonoids, during tomato fruit development, in parallel with an evaluation of regulatory genes would help elucidate the molecular basis of fruit nutritional quality. The expression of genes involved in metabolite accumulation during fruit development is known to vary between different species. For example, the L-galactose-1-phosphate phosphatase (*GGP*) gene which catalytic L-Galactose1-P to form L-Galactose was initially reported to have the highest expression during maturation, and was thus assumed to be the limiting step for ascorbic acid biosynthesis in tomato [7]. However, *GGP* expression showed a decreasing pattern in the ascorbic acid-rich fruit of chestnut rose, but the expression of the dehydroascorbate reductase (*DHAR*) gene which was the key enzyme involved in ascorbic acid recycling pathway correlated with ascorbic acid accumulation [45]. Such examples underline the complexity and diversity of the key pathways that determine metabolite levels. Some transcription factors that regulate metabolite biosynthesis have been isolated by map-based cloning or mutant screening, including MYB12, which regulates flavonoid biosynthesis in tomato [22], ERF98 which modulates ascorbic acid biosynthesis in Arabidopsis [18] and PIF1 which controls carotenoid biosynthesis in Arabidopsis [12]. However, correlation analysis of enzyme encoding genes and transcription factors also provides an effective approach to finding candidate transcription factors [12, 22]. Using transcriptional and metabolic association analysis, we identified transcription factors that are potentially associated with the biosynthesis of ascorbic acid, carotenoids and flavonoids during tomato development (Table 3). The expression of the transcription factor LIM (*Solyc06g071310*.*2*) is highly correlated with the expression of genes involved ascorbic acid and flavonoid biosynthesis (Table 3 and S4 Table) and also with the total ascorbate concentration in an introgression population derived from the wild tomato species *S*. *pennelli* (http://ted.bti.cornell.edu/cgi-bin/TFGD/array_data/probe_expression.cgi?array_ID=A03&probe_ID=LE1O19). A MYB transcription factor (*Solyc09g010840*.*1*) show a decreasing transcriptional patter during fruit development and ripening in AC fruit, while its transcription abundance in ripe fruit of HG6-61 is higher than that in AC (S3 Table). This transcription pattern is consistent with metabolite level of ascorbic acid. The ascorbic acid concentration in fruits of HG6-61 is higher than that in AC at 49 DAF (Fig 1A). These results support our hypothesis that the transcription factors listed in Table 3 are potentially involved in regulating ascorbic acid biosynthesis. We also identified a gene encoding *MYB12* (*Solyc01g079620*.*2*), which has been reported to be an important regulator of flavonoid biosynthesis in tomato fruit, and to be positively associated with the expression levels of 9 of 14 structural genes from the flavonoid biosynthesis pathway [22]. Moreover, the transcription abundance of *MYB12* in reaches very high level in green mature fruit stage (S3 Table), which is consistent to the high level of major flavonoid compound (e.g. naringenin chalcone) (Fig 1C). In our correlation analysis, expression of *MYB12* showed a high correlation with transcript levels of several flavonoid biosynthetic genes in the two tomato genotypes (Table 3 and S4 Table), while several other *MYB* and *bHLH* genes also showed a significant expression correlation with such genes, and may therefore also be involved in the regulation of flavonoid biosynthesis as previously report [46]. Likewise, three AUX/IAA genes (*Solyc03g120500*, *Solyc09g090910* and *Solyc12g007230*) were selected in ascorbic acid biosynthesis, consistent with previously report that ascorbic acid content in tomato fruit is associated with genes involved in hormone signaling [47]. Unexpectedly, regulators related to ripening and carotenoid accumulation such as RIN, CNR and Nor were not found in the correlation analysis (Table 3). It is most likely that RIN, CNR and Nor genes were co-expressed with individual genes involved in carotenoid biosynthesis, e.g. *PSY1* or *PDS1*, instead of major of biosynthetic genes.

Most metabolites showed similar fluctuations in the two cultivars during fruit development, but the accumulation of several compounds in carotenoids and flavonoids in AC fruits reached to its peak value earlier than in HG6-61, suggesting different ripening progress (Fig 1). This can also be reflected by the differential response to ripening at transcription level. The expression of *PSY1* showed a delayed activation in HG6-61 compared to AC (S3 Table). Also, the expression of *RIN* gene (*Solyc05g012020*) and *AP2* (*Solyc03g044300*) which were normally ripening induced were low in both cultivars during fruit enlargement stage (before 35 DAF), and were induced earlier in AC than in HG6-61. Although the ripening progress is slightly different between two genotypes, the focus of our study is on the correlation of transcription abundance of transcription factors and enzymatic genes or metabolites. We carried out correlation analysis in these two varieties respectively and the ripening variation will not affect our independent correlation analysis in each cultivar. Same transcription factors were selected by correlation analysis from these two different ripening genotypes even in some published data (Heizn1706 and *S*. *pimpinellifolium*) (S5 Table), revealing their conserved involvement in carotenoids, flavonoids and ascorbic acid metabolism.

Agroinfiltration provides a high throughput transient gene expression system that has been widely used in the gene functional analyses [48]. In this current study, this transient expression system was employed to investigate the influence of candidate transcription factors on metabolite pathway genes. The expression levels of the three transcription factors investigated here were significantly enhanced *in vivo* by agroinfiltration (S6 Fig) and, as a result, most of the structural genes that we surveyed in the various biosynthetic pathways were up-regulated as hypothesized, while expression of only a few of the genes was down-regulated or remained unchanged. This suggests that most of the selected transcription factors are involved in the regulation of these structural genes. One exception was *GMP2*, the expression of which was negatively correlated with the expression of *MYB* (*Solyc09g010840*) in the co-expression analysis (S4 Table), but was up-regulated when *MYB* was overexpressed (Fig 7). In contrast, the *APX5* gene was positively associated with *MYB* in the co-expression analysis (S4 Table), but was down-regulated when *MYB* was overexpressed (Fig 7), highlighting the complex regulatory network controlling metabolite biosynthesis. The co-expression analysis of transcription factors and enzyme encoding genes did not inherently indicate whether the identified transcription factors were potential inducers or suppressors of the regulated genes. If the transcription factors work downstream of the enzyme encoding genes, it is possible that a feedback inhibition would occur in the agroinfiltrated fruits to maintain metabolite levels. On the other hand, the expression levels of different members of a gene family may be differentially affected by transcriptional regulation, and the expression of the enzyme encoding genes may affect other genes involved in the biosynthetic pathway.

The RNA-seq transcriptome profiling provides an indication of the molecular mechanisms that govern fruit development at the transcriptional level. Processes that are known to be stage related were evident the patterns of gene expression: an example was the general down-regulation of photosynthetic light reactions during fruit development in the last two developmental stages (Fig 5), in agreement with the reduced photosynthesis capacity and carbon assimilation in ripe fruit [49]. The gene encoding the ethylene response factor 9 (*Solyc07g053740*.*1*) showed high expression levels particularly at later stages, suggesting that it may play an important role in fruit ripening (S3 Table), and its expression was also negatively correlated with flavonoid biosynthetic genes (Table 3), which are expressed earlier in development. However, there were also some inconsistencies between the gene expression and the corresponding biological process. The expression of genes encoding a pectinesterase, a pectate lyase and a xyloglucan endotransglucosylase hydrolase (*Solyc12g008530*.*1*, *Solyc03g071570*.*2* and *Solyc07g052980*.*2*) decreased throughout fruit development and was almost undetectable at the last two stages, which is consistent with previously reported results [44, 50] but contrary to previously reported patterns of cell wall degradation during fruit ripening [50].

In summary, the transcriptome of tomato fruit during development and ripening was extensively investigated. Correlations between the expression of metabolite biosynthetic genes and transcription factors were used to suggest candidate transcription factors that may regulate metabolite formation. Using RNA-seq analysis, the transcript abundance of a total of 26,397 genes was revealed. A total of 823 transcription factors were identified and their expression levels were compared to those of genes encoding enzymes involved in flavonoid, ascorbic acid and carotenoid biosynthesis. This revealed 20, 34 and 37 transcription factors putatively involved in the biosynthesis of flavonoids, ascorbic acid and carotenoids, respectively. Most of these candidate transcription factors have not previously been associated with metabolite biosynthesis, although functional evidence is available for a few, such as MYB12 [22]. Finally, three selected transcription factors (MYB, NAC and ZIF) were shown to modulate the expression levels of genes involved in the biosynthesis of ascorbic acid.

## Supporting Information

S1 Fig mRNA coverage analysis per hundred bins in Ailsa Craig and HG6-61 at different developmental stages. The numbers from one to seven indicate 7, 14, 21, 28, 35, 42 and 49 days after flowering (DAF), respectively, for Ailsa Craig (A) or HG6-61 (H).
(DOC)

S2 Fig mRNA expression profile reflected by RPKM in Ailsa Craig and HG6-61 at different developmental stages. The numbers from one to seven indicate 7, 14, 21, 28, 35, 42 and 49 DAF, respectively, for Ailsa Craig (A) or HG6-61 (H).
(DOC)

S3 Fig Transcriptome dynamics in HG6-61 during fruit development and ripening. The log_2_ values of reads per kilo base of a gene per million reads (RPKM) for each gene were used for the k-mean clustering analysis of seven developmental stages (7, 14, 21, 28, 35, 42 and 49 DAF). A total of 26,684 genes were grouped into 20 regulatory patterns, designated groups 1–20.
(DOC)

S4 Fig Correlation of gene expression between Ailsa Craig and HG6-61 during fruit development. The numbers from one to seven indicate 7, 14, 21, 28, 35, 42 and 49 DAF, respectively, for Ailsa Craig (A) or HG6-61 (H).
(DOC)

S5 Fig Total number of differentially expressed genes during fruit development in Ailsa Craig and HG6-61. Differentially expressed genes with RPKM fold changes of ≥2.0 or ≤0.5.
(DOC)

S6 Fig Expression of three selected transcription factors in agroinfiltrated fruits. Transcript levels of MYB (*Solyc09g010840*.*1*), NAC (*Solyc12g013620*.*1*) and ZIF (*Solyc06g065440*.*1*), the expression of which correlate with the expression levels of structural genes involved in the ascorbic acid biosynthesis pathway. AC means agroinfiltrated with empty vector. For each transcription factor two independent lines were selected. Results represent mean values (±SE) from three independent experiments.
(DOC)

S1 Table PCR primers used in this study. (DOC)

S2 Table The relative expression levels of 26,397 expressed tomato genes in Ailsa Craig and HG6-61, as determined by RNA-seq. (XLS)

S3 Table The RPKM values of 824 transcription factors used for co-expression analysis of structural genes involved in ascorbic acid, carotenoid and flavonoid metabolism. (XLS)

S4 Table Correlation analysis of transcription factors and structural genes. (XLS)

S5 Table Correlation analysis of selected transcription factors and structural genes in AC, HG6-61, Heizn1706 and the wild relative *Solanum pimpinellifolium*. (XLS)

S6 Table Correlation analysis between metabolite abundance and the transcript abundance of its related transcription factors and structural genes. (XLS)
