# Supplementary material for: Transcriptome Profiling of Tomato Fruit Development Reveals Transcription Factors Associated with Ascorbic Acid, Carotenoid and Flavonoid Biosynthesis
Source: PLoS One. 2015 Jul 2;10(7):e0130885. doi: 10.1371/journal.pone.0130885 (PMC4489915; doi:10.1371/journal.pone.0130885)
Supplement: S2 Fig — The numbers from one to seven indicate 7, 14, 21, 28, 35, 42 and 49 DAF, respectively, for Ailsa Craig (A) or HG6-61 (H). (DOC) [file pone.0130885.s002.doc]

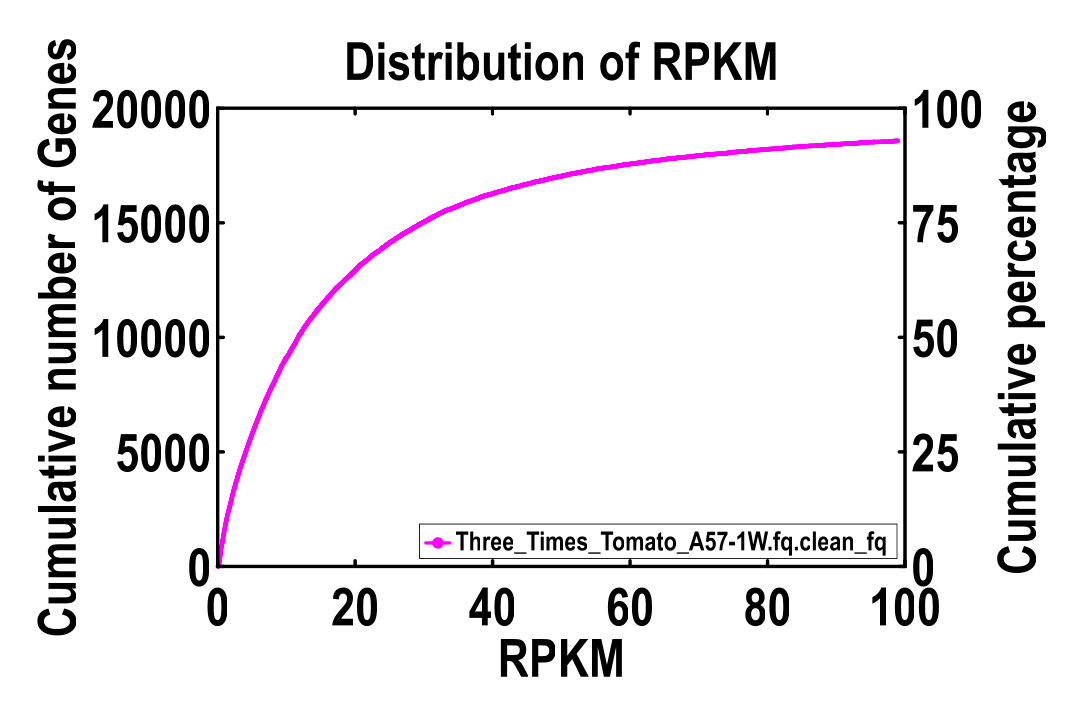

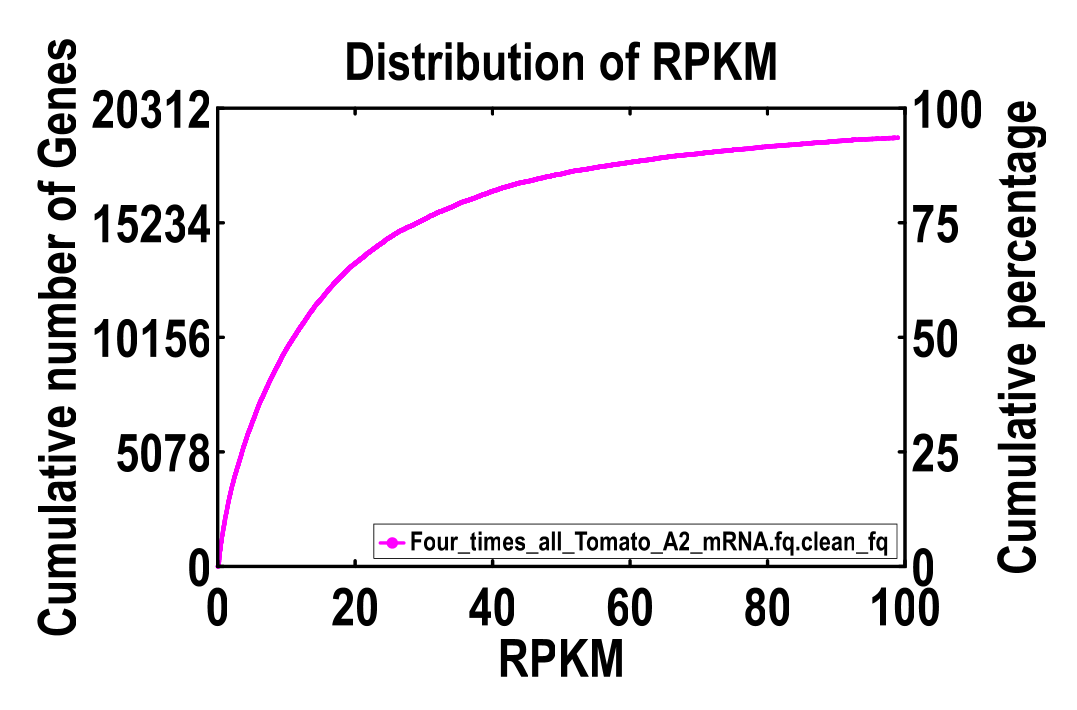

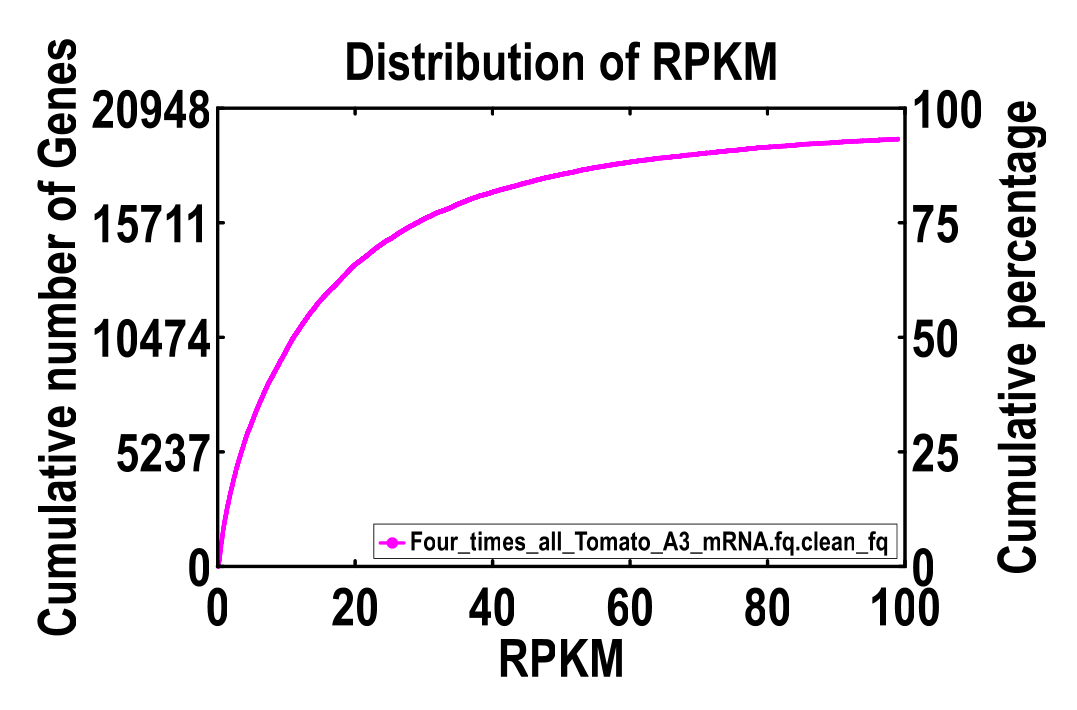

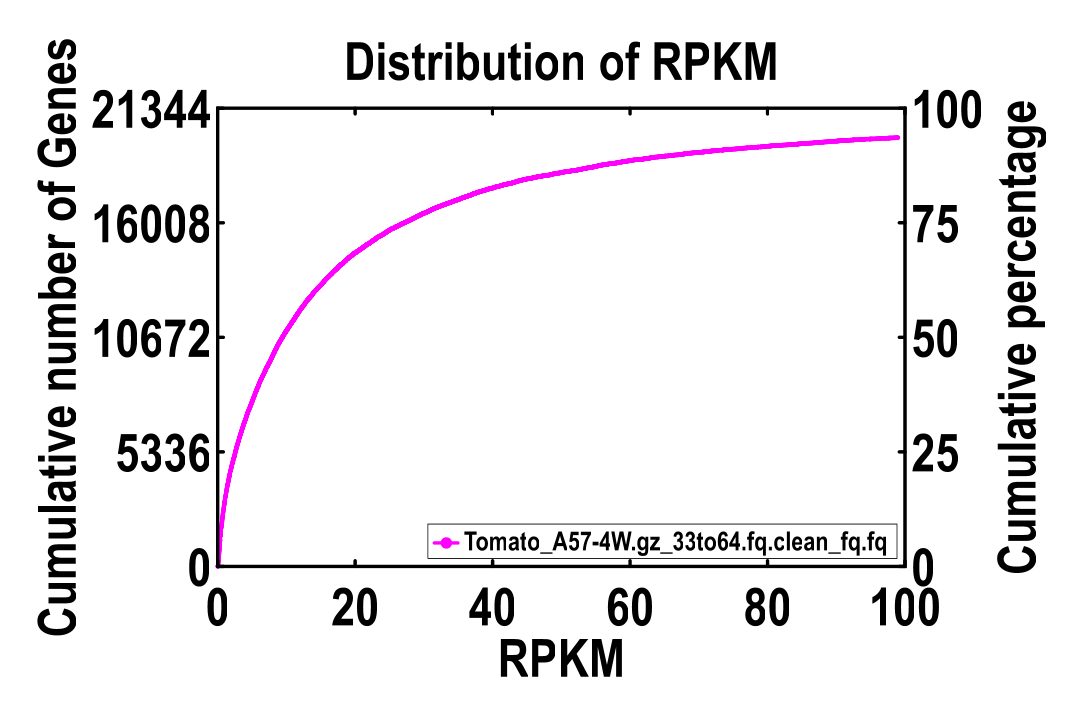

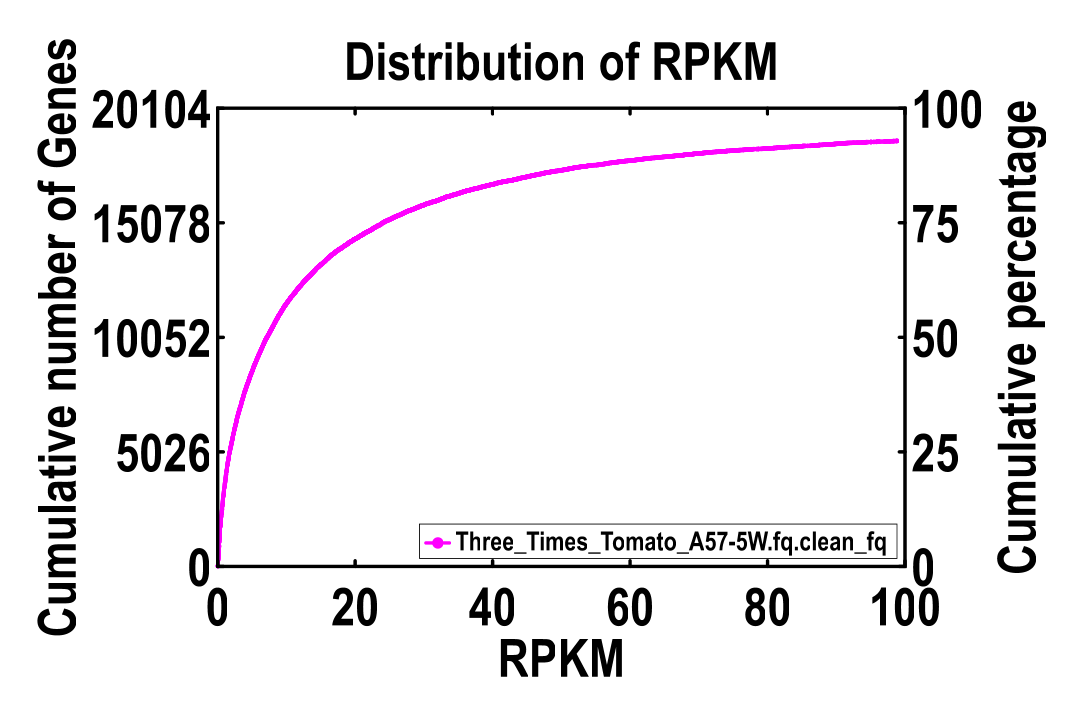

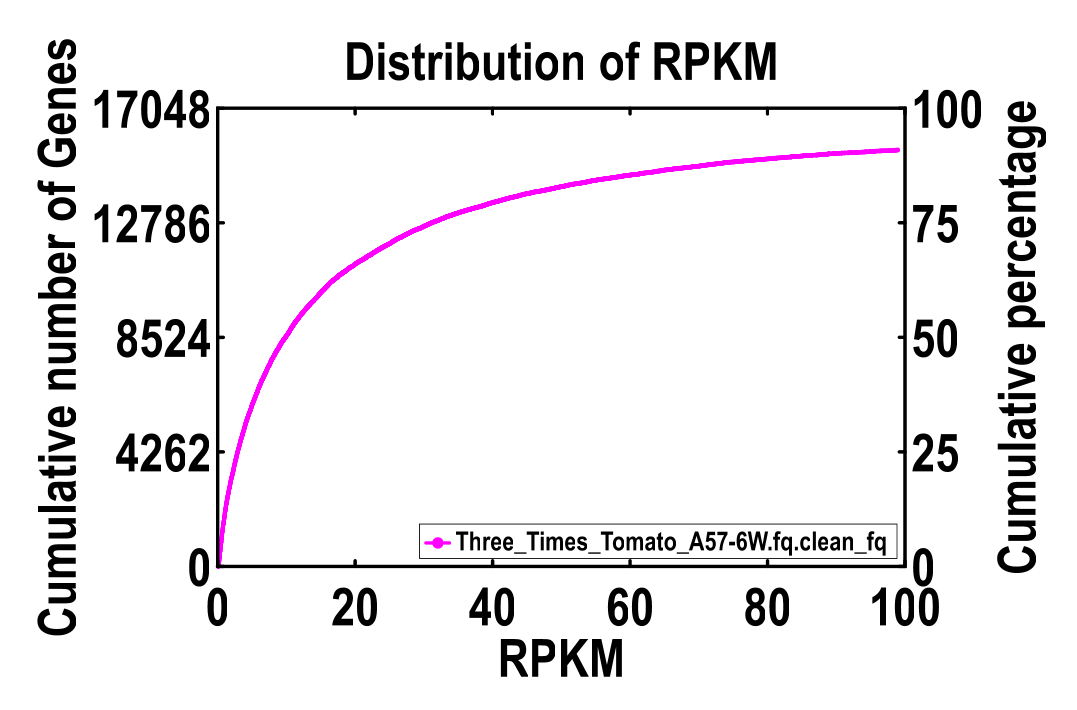

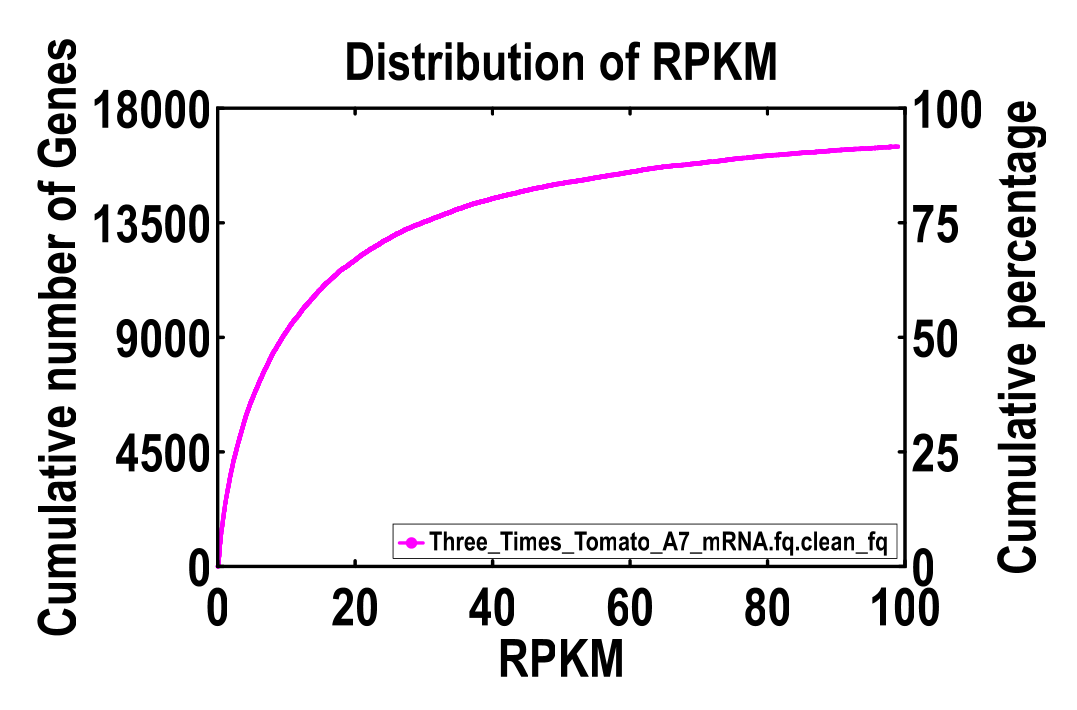

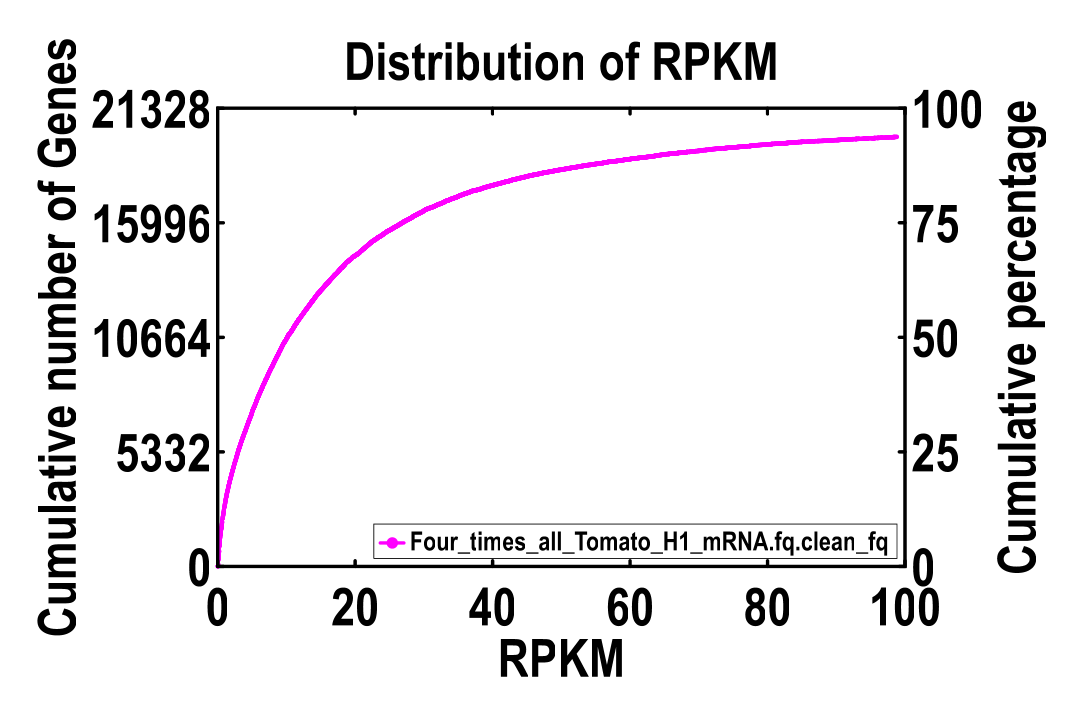

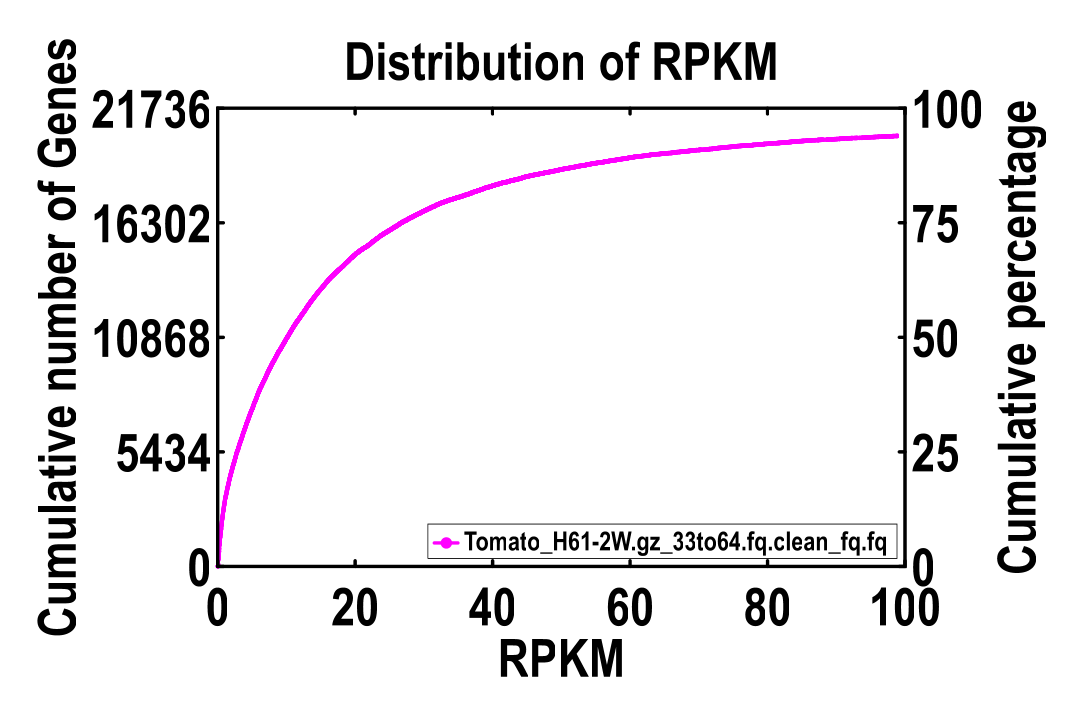

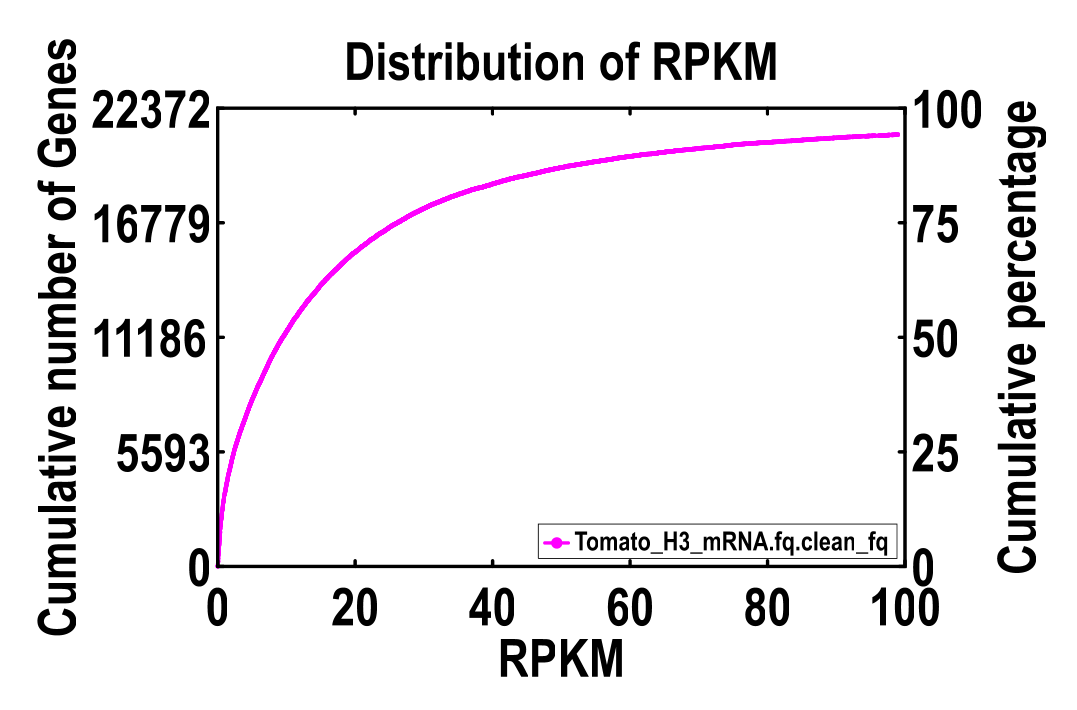

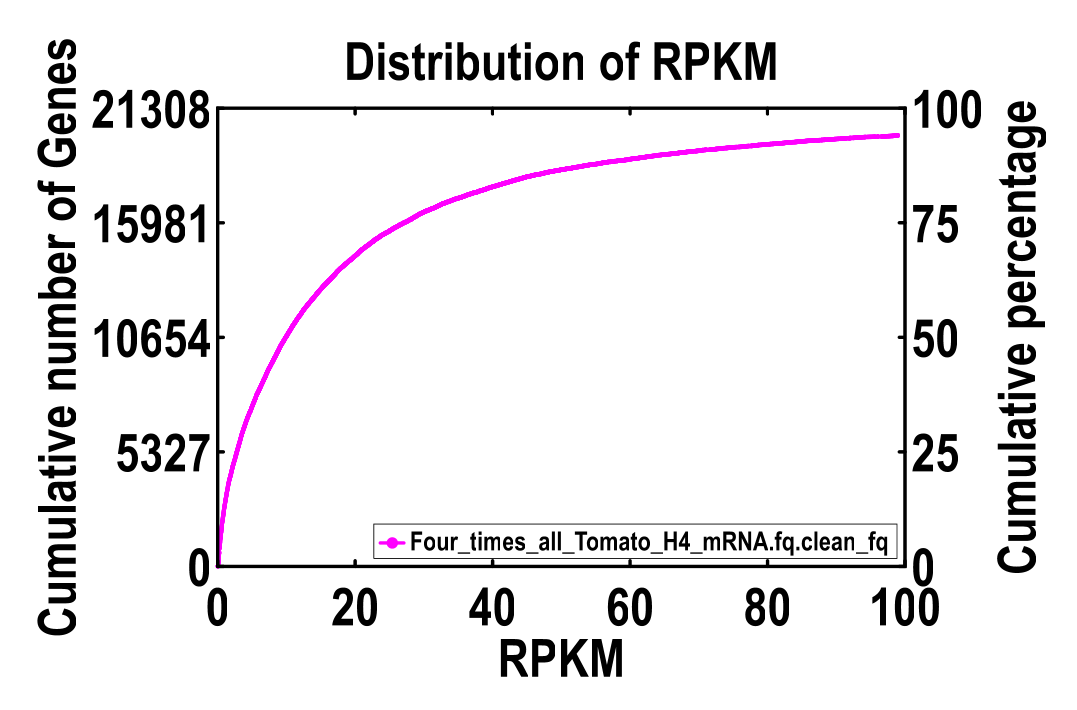

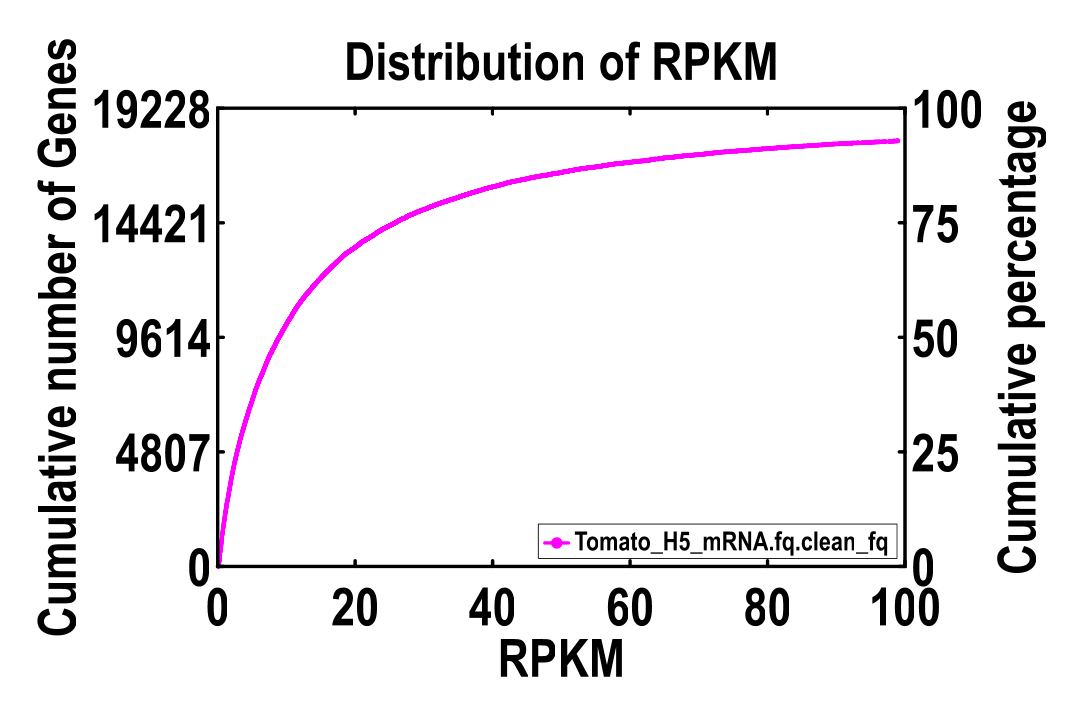

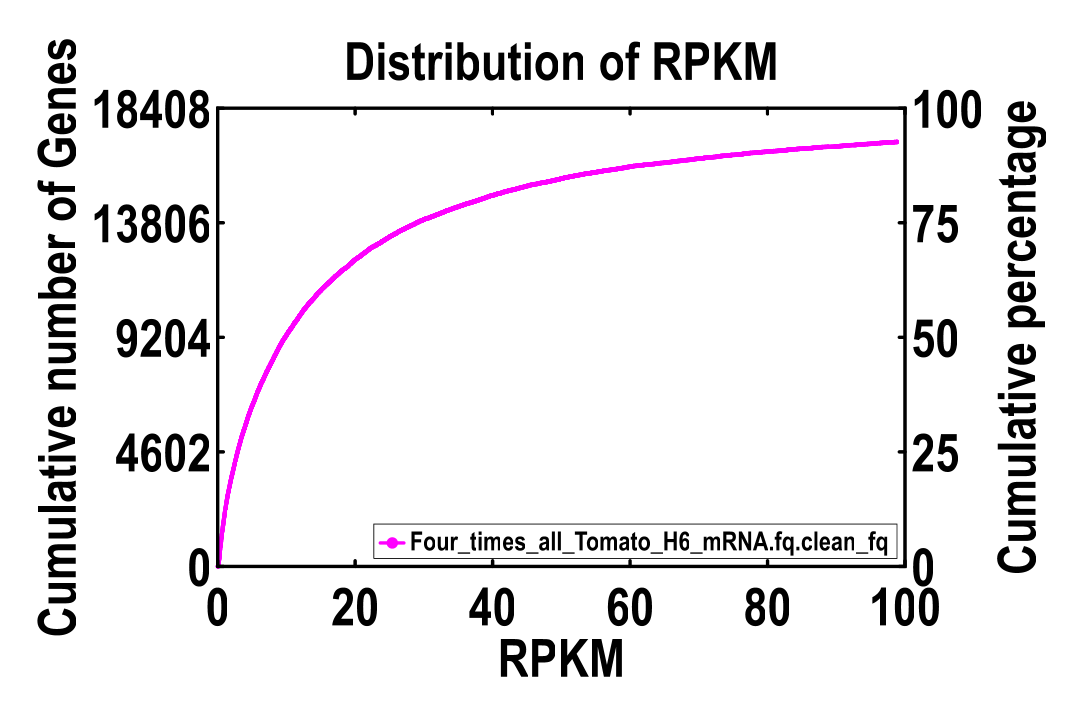

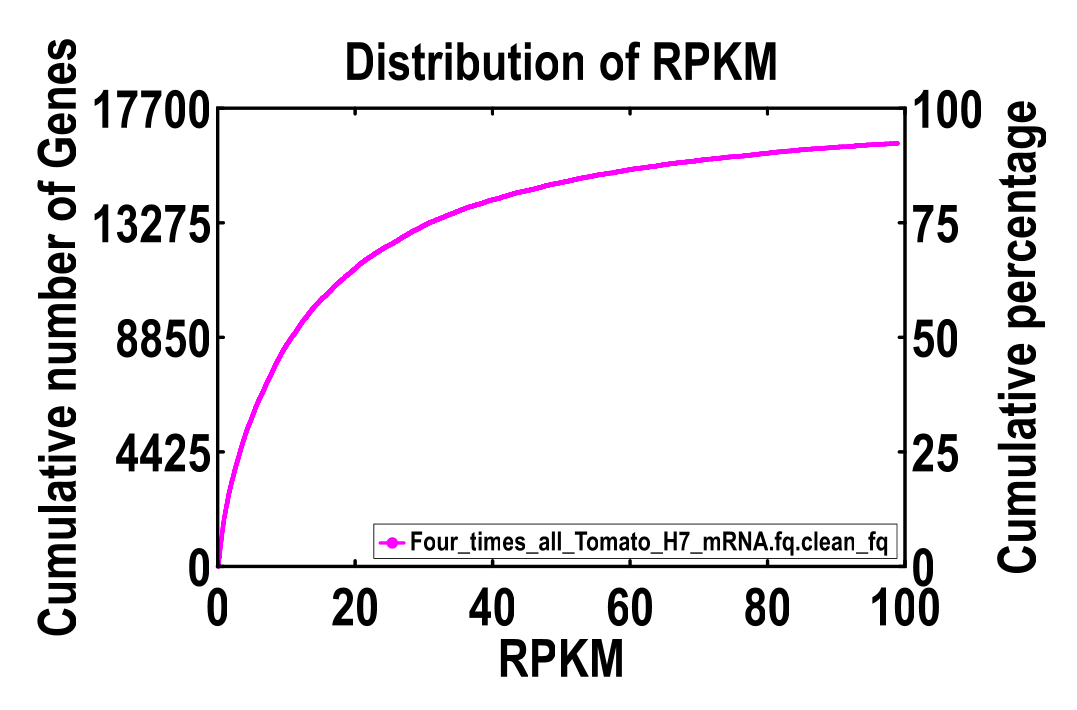


**A1**

**A2**

**A3**

**A4**

**A5**

**A6**

**A7**

**H1**

**H2**

**H3**

**H4**

**H5**

**H6**

**H7**

## Figure S2. mRNA expression profile reflected by RPKM in Ailsa Craig and HG6-61 at different developmental stages. The numbers from one to seven indicate 7, 14, 21, 28, 35, 42 and 49 DAF, respectively, for Ailsa Craig (A) or HG6-61 (H).
