# Supplementary material for: Transcriptome Profiling of Tomato Fruit Development Reveals Transcription Factors Associated with Ascorbic Acid, Carotenoid and Flavonoid Biosynthesis
Source: PLoS One. 2015 Jul 2;10(7):e0130885. doi: 10.1371/journal.pone.0130885 (PMC4489915; doi:10.1371/journal.pone.0130885)
Supplement: S3 Fig — The log2 values of reads per kilo base of a gene per million reads (RPKM) for each gene were used for the k-mean clustering analysis of seven developmental stages (7, 14, 21, 28, 35, 42 and 49 DAF). A total of 26,684 genes were grouped into 20 regulatory patterns, designated groups 1–20. (DOC) [file pone.0130885.s003.doc]

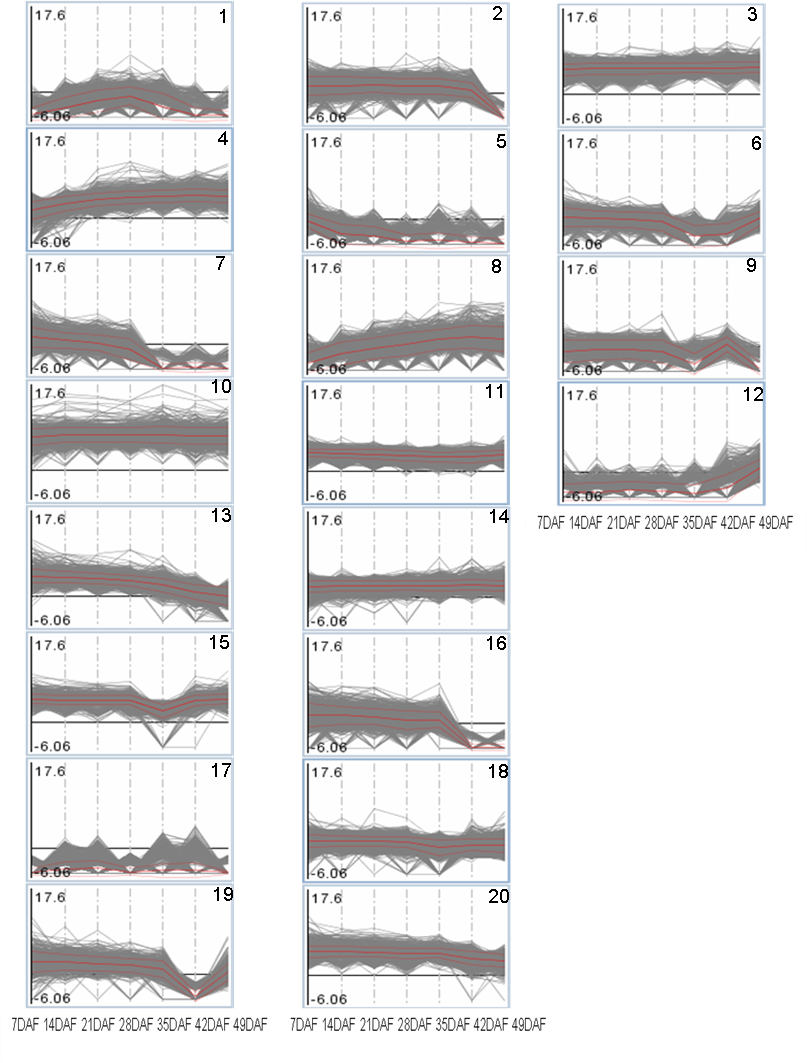

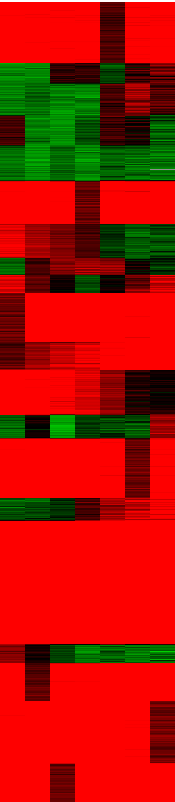

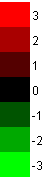

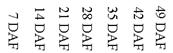


## Figure S3. Transcriptome dynamics in HG6-61 during fruit development and ripening. The log_2_ values of reads per kilo base of a gene per million reads (RPKM) for each gene were used for the k-mean clustering analysis of seven developmental stages (7, 14, 21, 28, 35, 42 and 49 DAF). A total of 26,684 genes were grouped into 20 regulatory patterns, designated groups 1-20.
