# Supplementary material for: Transcriptome Profiling of Tomato Fruit Development Reveals Transcription Factors Associated with Ascorbic Acid, Carotenoid and Flavonoid Biosynthesis
Source: PLoS One. 2015 Jul 2;10(7):e0130885. doi: 10.1371/journal.pone.0130885 (PMC4489915; doi:10.1371/journal.pone.0130885)
Supplement: S4 Fig — The numbers from one to seven indicate 7, 14, 21, 28, 35, 42 and 49 DAF, respectively, for Ailsa Craig (A) or HG6-61 (H). (DOC) [file pone.0130885.s004.doc]

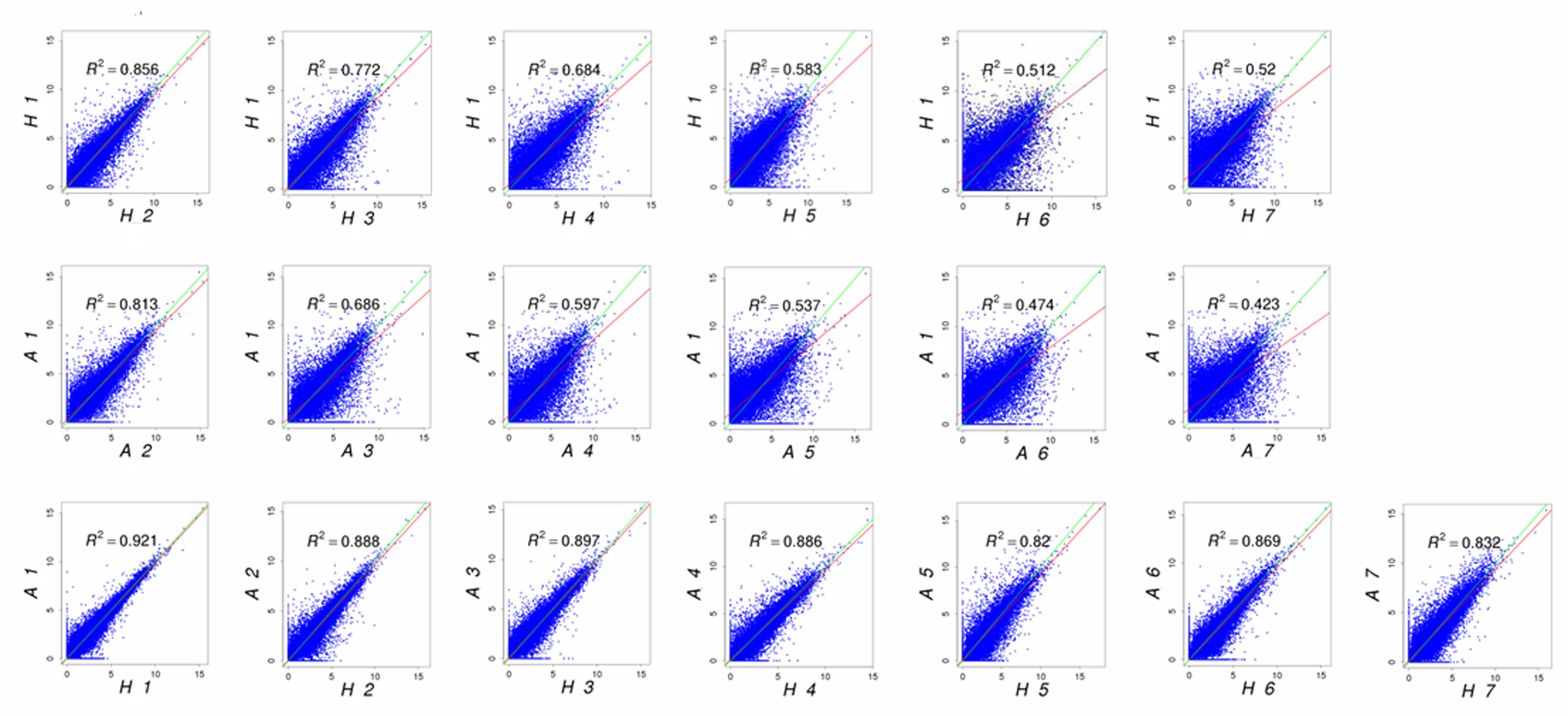


## Figure S4. Correlation of gene expression between Ailsa Craig and HG6-61 during fruit development. The numbers from one to seven indicate 7, 14, 21, 28, 35, 42 and 49 DAF, respectively, for Ailsa Craig (A) or HG6-61 (H).
