# Supplementary material for: Transcriptome Profiling of Tomato Fruit Development Reveals Transcription Factors Associated with Ascorbic Acid, Carotenoid and Flavonoid Biosynthesis
Source: PLoS One. 2015 Jul 2;10(7):e0130885. doi: 10.1371/journal.pone.0130885 (PMC4489915; doi:10.1371/journal.pone.0130885)
Supplement: S5 Fig — Differentially expressed genes with RPKM fold changes of ≥2.0 or ≤0.5. (DOC) [file pone.0130885.s005.doc]

## Figure S5. Total number of differentially expressed genes during fruit development in Ailsa Craig and HG6-61. Differentially expressed genes with RPKM fold changes of ≥2.0 or ≤0.5.
