# Supplementary material for: Transcriptome Profiling of Tomato Fruit Development Reveals Transcription Factors Associated with Ascorbic Acid, Carotenoid and Flavonoid Biosynthesis
Source: PLoS One. 2015 Jul 2;10(7):e0130885. doi: 10.1371/journal.pone.0130885 (PMC4489915; doi:10.1371/journal.pone.0130885)
Supplement: S6 Fig — Transcript levels of MYB (Solyc09g010840.1), NAC (Solyc12g013620.1) and ZIF (Solyc06g065440.1), the expression of which correlate with the expression levels of structural genes involved in the ascorbic acid biosynthesis pathway. AC means agroinfiltrated with empty vector. For each transcription factor two independent lines were selected. Results represent mean values (±SE) from three independent experiments. (DOC) [file pone.0130885.s006.doc]

## Figure S6. Expression of three selected transcription factors in agroinfiltrated fruits. Transcript levels of MYB (*Solyc09g010840.1*), NAC (*Solyc12g013620.1*) and ZIF (*Solyc06g065440.1*), the expression of which correlate with the expression levels of structural genes involved in the ascorbic acid biosynthesis pathway. AC means agroinfiltrated with empty vector. For each transcription factor two independent lines were selected. Results represent mean values (±SE) from three independent experiments.
