# Supplementary material for: Transcriptome Profiling of Tomato Fruit Development Reveals Transcription Factors Associated with Ascorbic Acid, Carotenoid and Flavonoid Biosynthesis
Source: PLoS One. 2015 Jul 2;10(7):e0130885. doi: 10.1371/journal.pone.0130885 (PMC4489915; doi:10.1371/journal.pone.0130885)
Supplement: S1 Table — (DOC) [file pone.0130885.s007.doc]

## Table S1. PCR primers used in this study.

| **Gene name** | **Gene accession** | **Forward** | **Reverse** | **Usage** |
| --- | --- | --- | --- | --- |
|  | Solyc05g006650 | AGTCTCCGCCGTTTAATTTATCTG | ATTATTCCGAACATCATCACTCACA | qRT-PCR confirmation of RNA-seq |
|  | Solyc01g079620 | AGGCCAAGGGAAGCCTTAGTT | TTATCTTCATCATCAATCACCACGT |
|  | Solyc01g087030 | TTCGTCAGCTCCAGCTCAACA | CCAAATACTCGGAAGAAATGCC |
|  | Solyc06g071310 | GGCAACTTCAGCCAAATGGA | AATGTTCACGGCTAGGCTCGT |
|  | Solyc04g078420 | AAGCCAACCCAGTCCCCTAA | ATGACCCATGAAAGCCGATG |
|  | Solyc02g084630 | AGGGGAAGACATGAGCGGACT | TCGTTCACGAATCTCAGCAACA |
|  | Solyc04g049670 | CTCAGAGTTCGTGGTCTAAAAGGG | AGTGCTTCTGGTCTTGAATGGATAA |
|  | Solyc01g096050 | CCATTTGTGAACGCACGGAA | GGGGTTGGAGGAGCATAGAGC |
|  | Solyc12g013620 | CTCCAGGCGTTCAGGGTCA | GGCGTAAACATCGTTGAGAAAAT |
|  | Solyc09g010840 | AGGGGAAGACATGAGCGGACT | TCGTTCACGAATCTCAGCAACA |
|  | Solyc05g007180 | TCTGGTAGTAGAGGGATCATGGATT | GATCTCCTCATTAACATGGATGGTA |
|  | Solyc12g007230 | TACTTTCGCTGCAAACATTCAA | TCCTGGCGTCGATCTTCAA |
|  | Solyc10g078700 | GCTGCTAGTGAGCTTGTTTTGC | ATCCCCAGGACCGATTTGA |
|  | Solyc02g037530 | AGGGGAAATTGTAGCTTGGATT | GTGGTCGGAAGGAGATGCC |
|  | Solyc06g073980 | CGCCGCCTCTGTCAGTTATC | GAGGTAGAAGCATTGGGTAGGG |
|  | Solyc12g013620 | AAAAAGCAGGCTTATGGGTGTTCAAGAAAAAGATCC | AGAAAGCTGGGTCTGGCATTCAGATTTCGAGCTA | amplification of full-length cDNA. |
|  | Solyc06g065440 | AAAAAGCAGGCTATGGAACAACAGGCTAATCAAAG | AGAAAGCTGGGTCTATATTTCCAGCCTTTGTAGTTGC |
|  | Solyc09g010840 | AAAAAGCAGGCTTATGAGGAGTTTGGGAGGATGG | AGAAAGCTGGGTGAAAAGTCGCGGCAAGTAGAA |
|  | Solyc06g060100 | CTATGAAACGCTGGAAAGTGCC | ATTCCTGACCAATGCGGCC | qRT-PCR after agroinfiltration |
|  | Solyc08g008670 | TCAGTACGCAAAGTGCCACCG | TGCAGCCATTTCTGTTTTATCTTTC |
|  | Solyc06g051270 | TCCTAAAACCAGAAATAGTGATGTGAA | GCTATTCTTCAGCTTATTGCCTTACA |
|  | Solyc10g079470 | CCTTCTCCACCACCACTACCG | CCGAGAATGGAAAGGAATAATACG |
|  | Solyc08g016170 | CGTGTTTTCTGACTTCAAGCCATG | AACAAAAACAACCAAAATTCGCAA |
|  | Solyc02g070790 | GGCCACAACTCTGTCGTTGC | CCACACGGAGATGAAACAGACA |
|  | Solyc09g097970 | ATACAAAACAATATCCACTTGCTCAAT | TTGATTCTTCCCACTTTTGTTCC |
|  | Solyc11g062440 | CTATTGCACCCCTTTGGAATGATTA | TTGTGTAACCATCGATTTTATTCTGA |
|  | Solyc04g074640 | ATGGTCTCTTTTGCTTCAACACTTC | AAATCATACCAGGTTGAGGAGGG |
|  | Solyc02g087610 | TTCAACGCAACACTTACGCCG | GGGAGTCTGAACCTGGGCAAA |
|  | Solyc08g081530 | TTTGGTGACAATGTTGGGGAG | CCACTTTCAAGAAGAACCCCC |
|  | Solyc05g054760 | TGAAGTTTGTGTCAAGGCTGCTGT | AGTCACTTTCTTTTCCTCCAATGTC |
|  | Solyc01g097340 | AATCCGACTTCCGTGAGCC | CTGAGTTGCGACCACGGAC |
|  | Solyc09g082990 | CCATCACATTCCAGGACCAGA | CGTAATCCTCAACCCATCCTT |
|  | Solyc01g106450 | CTTCTTACTGAGGCTGGTGGTC | AACCTCTTTAACAGACTTCATCCC |
|  | Solyc06g005160 | TGGAGCCCATTAGGGAGCA | GCCAGGGTGAAAGGGAACAT |
|  | Solyc06g005150 | TGGGAGGGTGGTGACATATTTT | TTGAAGTGCATAACTTCCCATCTTT |
|  | Solyc09g007270 | TTCAACAGCAACTACTCCAGCC | GGAACAGTTCCCAATCCTATCC |
|  | Solyc02g083620 | AGTAGATGCAGAGTATCTGAAGGA | CATAGGTTCCTGCATCATGCCACC |
|  | Solyc11g018550 | CATGCCAAACTGAGCAACCTT | CCTCCCAAGCCTTCGTATTCT |
